# Hsa_circ_0003945 promotes progression of hepatocellular carcinoma by mediating miR‐34c‐5p/LGR4/β‐catenin axis activity

**DOI:** 10.1111/jcmm.17243

**Published:** 2022-02-16

**Authors:** Li‐Hua Lyu, Chun‐Yan Zhang, Wen‐Jing Yang, An‐Li Jin, Jie Zhu, Hao Wang, Te Liu, Bei‐Li Wang, Jian‐Wen Cheng, Xin‐Rong Yang, Wei Guo

**Affiliations:** ^1^ Department of Laboratory Medicine Zhongshan Hospital Fudan University Shanghai China; ^2^ Department of Laboratory Medicine, Xiamen Branch Zhongshan Hospital Fudan University Xiamen China; ^3^ Shanghai Geriatric Institute of Chinese Medicine Shanghai University of Traditional Chinese Medicine Shanghai China; ^4^ Cancer center, Zhong Shan Hospital Fudan University Shanghai China; ^5^ Department of Liver Surgery & Transplantation Liver Cancer Institute Zhongshan Hospital Fudan University Shanghai China; ^6^ Key Laboratory of Carcinogenesis and Cancer Invasion Ministry of Education Shanghai China; ^7^ Department of Laboratory Medicine, Wusong Branch Zhongshan Hospital Fudan University Shanghai China

**Keywords:** Circ_0003945, hepatocellular carcinoma, leucine‐rich repeat‐containing G protein‐coupled receptor 4, miR‐34c‐5p, tumour progression

## Abstract

Accumulating evidence suggests that circular RNAs (circRNAs) play essential roles in regulating cancer progression, but many circRNAs in hepatocellular carcinoma (HCC) remain unknown. Dysregulated circRNAs in HCC were identified through bioinformatics analysis of Gene Expression Omnibus data sets. Quantitative real‐time PCR (qRT‐PCR), Sanger sequencing, RNase R digestion and actinomycin D treatment were conducted to confirm the characterization of circRNAs. CCK‐8, wound‐healing and Transwell assays were performed to assess the functional roles of Hsa_circ_0003945 (Circ_0003945) in HCC cell lines. Subcellular fractionation and fluorescence in situ hybridization (FISH) were performed to locate Circ_0003945 in HCC cells. Dual‐luciferase reporter assay was executed to verify the binding of Circ_0003945 to microRNAs (miRNAs) or the miRNAs to their target genes. In this study, we found that Circ_0003945 was upregulated in HCC tissue, and higher Circ_0003945 expression was positively correlated with tumour size and tumour stage. Furthermore, high plasma levels of circulating Circ_0003945 were confirmed in HCC patients compared with those in non‐HCC groups. The functional experiments revealed that overexpression or knockdown of Circ_0003945 promoted or attenuated tumour growth and migration, respectively. Mechanistically, Circ_0003945 might exert as a miR‐34c‐5p sponge to upregulate the expression of leucine‐rich repeat‐containing G protein‐coupled receptor 4 (LGR4), activating the β‐catenin pathway, and finally facilitating HCC progression. Additionally, a β‐catenin activator could reverse the effect of Circ_0003945 knockdown. In conclusion, Circ_0003945 exerts a tumour‐promoting role in HCC cells by regulating the miR‐34c‐5p/LGR4/β‐catenin axis, which may be a potential target for HCC therapy.

## 1 INTRODUCTION

Hepatocellular carcinoma (HCC) is one of the most common malignant tumours globally.^1^ Surgery remains the most effective treatment strategy, with curative potential in well‐selected candidates.^2^ Despite improved surveillance and treatment strategies in recent years, the clinical outcome of HCC remains dismal due to the high rates of relapse and metastasis, which result in a poor 5‐year survival rate.^3^, ^4^ Thus, a thorough investigation into the mechanisms underlying HCC progression and metastasis is urgently needed to develop new therapeutic approaches and improve the clinical outcome of HCC patients.

CircRNAs are generated via proactive back‐splicing of pre‐mRNA and are endowed with neither 5’ to 3’ polarity nor a polyadenylated tail, but they are characterized by covalently closed‐loop structures and show resistance to ribonuclease R (RNase R) digestion.^5^, ^6^, ^7^ Accumulating evidence indicates that circRNAs are abnormally expressed in many types of cancers and are closely related to cancer progression and prognosis.^8^, ^9^, ^10^ Furthermore, circRNAs might mediate pathological processes of cancer cells,^11^, ^12^ such as acting as a miRNA sponge to mediate the biological function of their downstream targets or regulate their parental gene expression.^9^, ^13^ Although some aberrant circRNAs play important roles in HCC tumorigenesis and progression,^14^, ^15^, ^16^ the overall biological and molecular contributions of most circRNAs to HCC progress remain elusive.

In the present study, dysregulated circRNAs in HCC were screened by analysing Gene Expression Omnibus (GEO) datasets followed by verification in clinical samples. We further characterized one significantly overexpressed circRNA derived from exons 11 and 12 of the ubiquitin‐associated protein 2 (UBAP2) and named Hsa_circ_0003945 (Circ_0003945) in the circBase database. The function and mechanism of Circ_0003945 in HCC progression were also investigated.

## 2 MATERIALS AND METHODS

### 2.1 Methods for screening circRNA profiles of HCC tissue

To show the dysregulated circRNAs, GEO databases (https://www.ncbi.nlm.nih.gov/geo/; GSE78520, GSE94508 and GSE97332) were selected based on inclusion of results of circRNA microarray analysis of HCC tissue and paired healthy adjacent liver tissue.^17^, ^18^ The GEO2R tool was used to screen the dysregulated circRNAs in HCC tissue, with a *p* value <0.05 used as a cut‐off threshold.

### 2.2 Clinical specimens

All human HCC tissue and paired adjacent normal liver tissue (*n* = 50) and plasma samples (*n* = 151, including 41 cases of healthy donors [HD], 40 cases of chronic liver hepatitis [CHB], 30 cases of liver cirrhosis [LC] and 40 cases of HCC) were collected from the Zhongshan Hospital of Fudan University from September 2018 to December 2020 and stored at −80°C until analysis. The criteria for diagnosing CHB and LC were consistent with our previous work.^19^ The final diagnosis of HCC was verified histologically by two pathologists. The present study was approved by the Medical Ethics Committee of Zhongshan Hospital, Fudan University. All participants in this study provided written informed consent in accordance with the Declaration of Helsinki.

### 2.3 Cell lines and cell culture

LO2 normal liver cells, HepG2 hepatoma cells and HCC cell line, MHCC97L, MHCC97H and HCCLM3 were obtained from the Liver Cancer Institute, Fudan University (Shanghai, China). SUN387, SK‐Hep1, Li‐7, Hep3B and human embryonic kidney (HEK‐293T) cell lines were purchased from the cell bank at the Institute of Biochemistry and Cell Biology, China Academy of Science (Shanghai, China). All cells were cultured in high‐glucose Dulbecco's modified Eagle medium (DMEM; Cat. 10566016, Gibco) supplemented with 10% foetal bovine serum (FBS, Cat. BS‐0003‐500, BioSUN), 1% penicillin, and 100 μg/ml streptomycin. Cells were cultured in a humidified atmosphere of 5% CO_2_ at 37°C. Further experimental methods *in vitro* were shown in supplementary materials and methods.

### 2.4 Animal experiments

Male BALB/c nude mice (5–6 weeks old) were purchased from the Department of Experimental Animals of the Chinese Academy of Sciences (Shanghai, China). HCCLM3 cells transfected with shCirc_0003945 or shNC (5 × 10^6^ cells/mouse) were injected subcutaneously into the right dorsum to generate subcutaneous tumours (*n* = 6/group). Mouse body weight and tumour size were measured every week. Four weeks later, mice were sacrificed and tumours were weighed and processed for histological analysis. Tumour volume was calculated according to the formula: volume (mm^3^) = width^2^ × length/2. All animal care and procedures were performed following guidelines approved by the Institutional Animal Care and Use Committee at Zhongshan Hospital, Fudan University.

### 2.5 Statistical analysis

Statistical analysis was performed using Statistical Program for Social Sciences (SPSS) 24.0 Software (SPSS) and GraphPad Prism 8.0 (GraphPad Software). The distribution of each group was determined by the Kolmogorov‐Smirnov test. Student's *t*‐test (two‐tailed) was used to assess statistical significance between the two groups. The paired *t*‐test was used to analyse the statistical significance of circRNAs, miRNAs, or mRNAs between HCC and adjacent normal liver tissue. Chi‐square test was used to analyse the correlation between circRNA level and clinicopathological features of HCC patients. Pearson's correlation or Spearman correlation was used to assess the correlation between circRNA and miRNA or mRNA. All experiments were repeated at least three times. Data are presented as mean ±SD. *p* < 0.05 was considered statistically significant.

## 3 RESULTS

### 3.1 Characteristics and expression of Circ_0003945 in HCC tissue and cell lines

**FIGURE 1:**
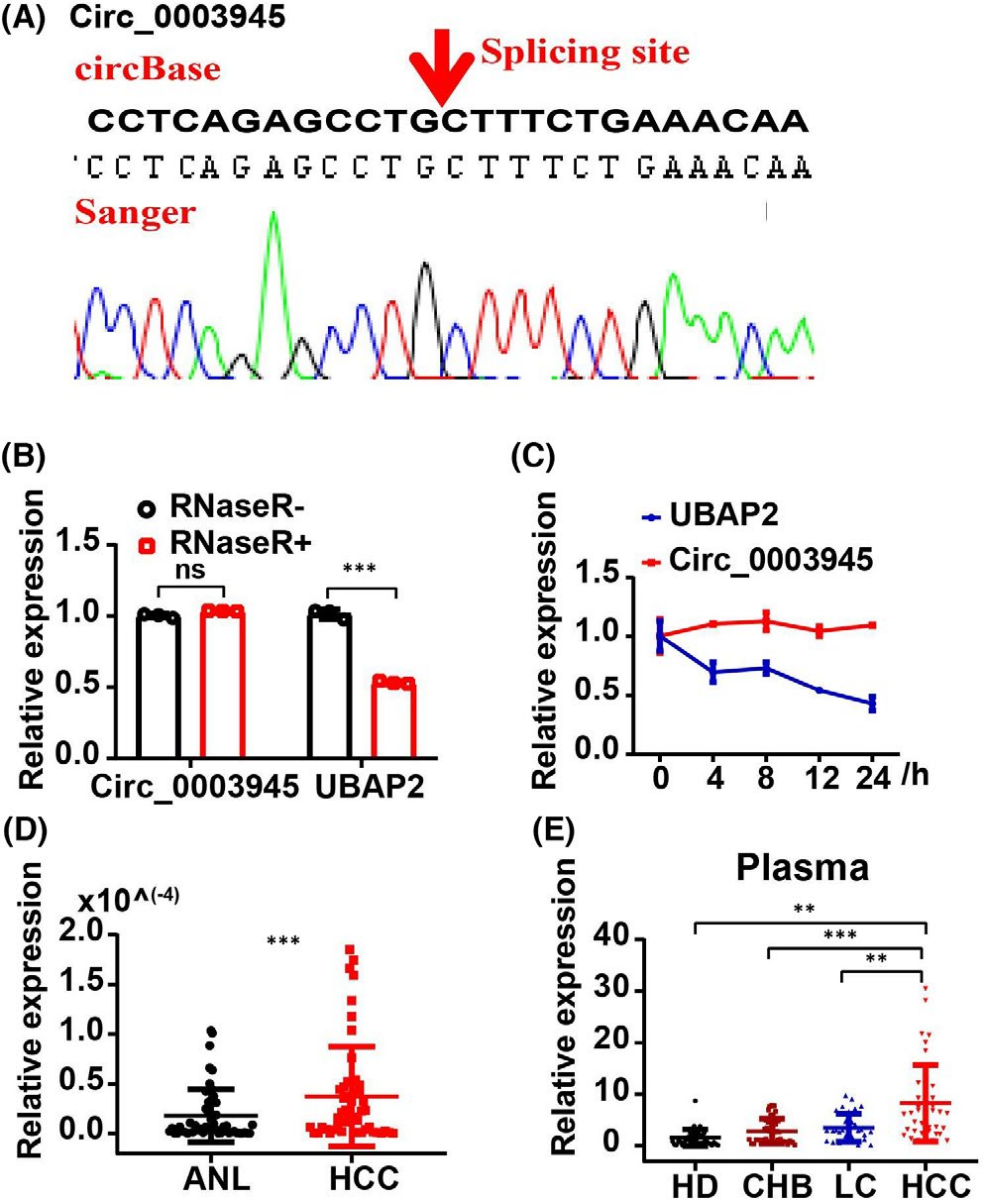
Characteristics and expression of Circ_0003945 in HCC samples. (A) Sanger sequencing of the qPCR product. Red arrow indicates the back‐splicing sites of Circ_0003945. (B) The qPCR analysis of Circ_0003945 and associated UBAP2 in HCC cells treated with or without RNase R. (C) Expression levels of Circ_0003945 and UBAP2 mRNA was determined in HCC cells treated with actinomycin D at indicated time points. (D) Expression level of Circ_0003945 was analysed by qPCR in adjacent neighbouring liver (ANL) and HCC tissue; paired t‐test was used. (E) Relative expression level of circulating Circ_0003945 in plasma; non‐parametric test was used. The RNase R treatment and actinomycin D experiments were performed in triplicate and t‐tests were used. (**p < 0.01; ***p < 0.001)

To screen dysregulated circRNA expression profiles in HCC tissue, we re‐analysed the datasets from the GEO database (Figure S1A). Ten significant candidate circRNAs were selected for further validation according to the *p* value (*p* < 0.05), |Log_2_FC| > 1.5 (Table S1). Finally, Hsa_circ_0001955 (Circ_0001955), Hsa_circ_0005397 (Circ_0005397), Hsa_circ_0027478 (Circ_0027478) and Hsa_Circ_0003945 (Circ_0003945) were successfully verified. The back‐spliced regions of these circRNAs were confirmed by Sanger sequencing, and all were in agreement with circBase (Figure  and Figure S1B). Furthermore, RNase R digestion revealed that candidate circRNAs were more resistant than associated mRNAs (Figure 1B and Figure S1C), and actinomycin D treatment clearly attenuated the half‐life of parental mRNA levels in HCC cells, but had little effect on circRNAs (Figure 1C and Figure S1D). These results confirmed the circular structure of circRNAs, showing that circRNAs were more stable than their linear forms.

Then, the expression level of circRNAs was measured by qPCR in HCC samples. We found that Circ_0001955, Circ_0005397 and Circ_0003945 were significantly increased in HCC tissue compared with adjacent normal liver tissue (Figure 1D and Figure S2A‐2B), with Circ_0003945 showing the most significant increase of the differentially expressed circRNAs (*p* < 0.001, Figure 1D); no significant difference was found in the transcription of Circ_0027478 (Figure S2C). Further analysis showed that higher expression level of Circ_0003945 in HCC tissue was closely associated with tumour size and China liver cancer staging (CNLC) stage, implying a possible role of Circ_0003945 in HCC progression (Table 1). Moreover, circulating Circ_0003945 in plasma was also significantly increased in HCC patients compared with non‐HCC groups (HD, CHB and LC; Figure 1E), indicating its potential diagnostic role.

**TABLE 1:** Correlations between Circ_0003945 expression level and clinicopathological characteristics in HCC

Clinicopathological characteristics	Circ_0003945		
	Low	High	*p* value
	25	25	
Age			0.156
<60	9	14	
≥60	16	11	
Gender			0.462
Male	19	22	
Female	6	3	
Liver cirrhosis			0.777
Yes	12	13	
No	13	12	
Tumour number			0.247
Solidarity	19	23	
Multiple	6	2	
Tumour Size (cm)			0.009
≤5	14	5	
>5	11	20	
Tumour encapsulation			
Complete	10	9	0.771
None	15	16	
Tumour differentiation			0.156
I‐II	14	9	
III‐IV	11	16	
Microvascular invasion			0.544
No	7	9	
Yes	18	16	
CNLC			0.023
I	15	7	
II+III	10	18	
AFP (ng/ml)			0.556
<20	10	8	
≥20	15	17	

Note

CNLC, China liver cancer staging; AFP, alpha fetoprotein.

### 3.2 Circ_0003945 promotes the proliferation and migration of HCC cells *in vitro*

**FIGURE 2:**
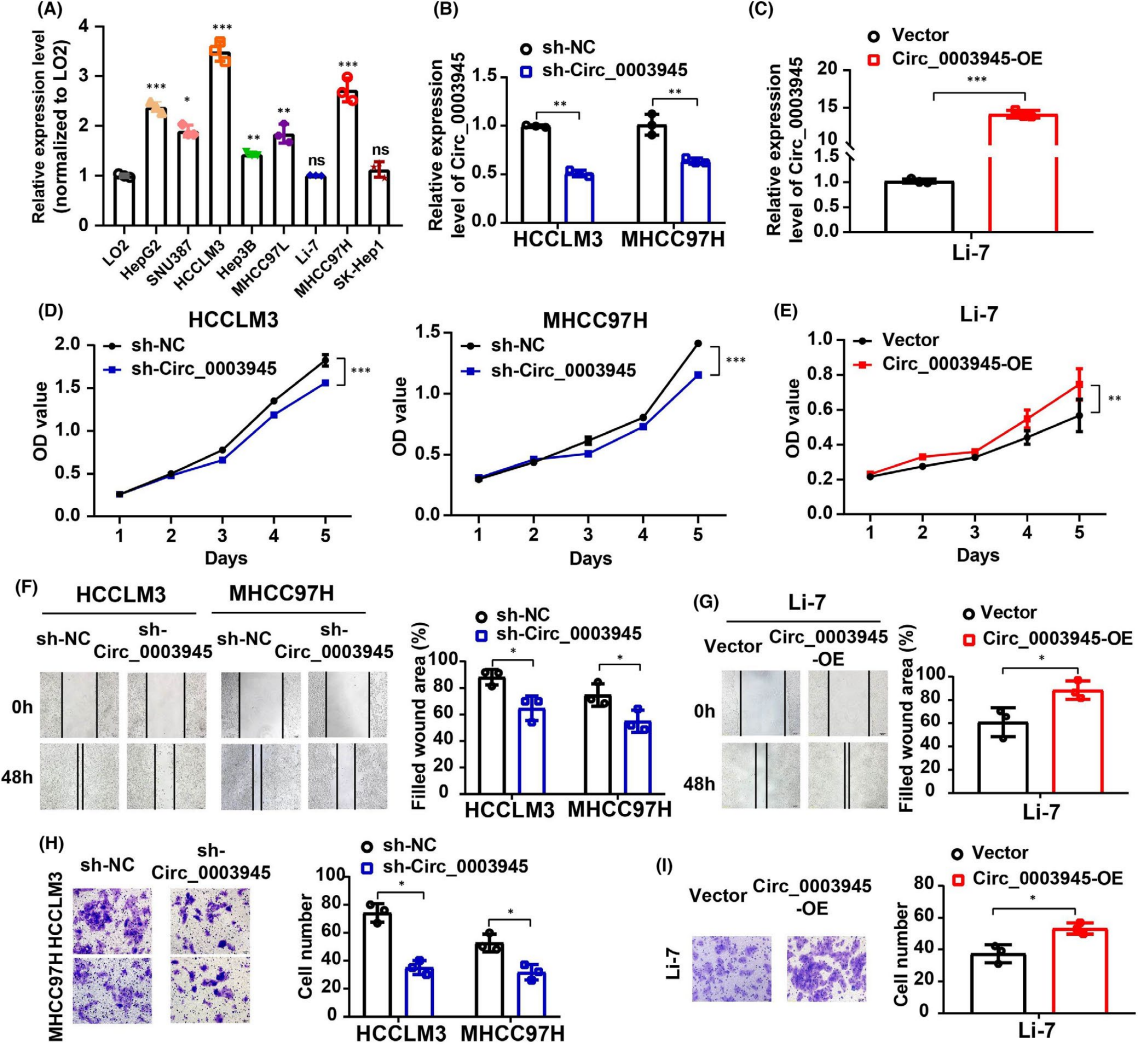
Effects of Circ_0003945 on proliferation, migration and invasion of HCC cells. (A) Relative expression level of Circ_0003945 in LO2 normal hepatocytes and HCC cell lines. (B and C) Expression of Circ_0003945 in stably transfected HCC cells. (D and E) Effect of Circ_0003945 knockdown or overexpression on the proliferation of HCC cells was monitored via CCK‐8 assays. (F and G) Migratory ability was assessed by wound‐healing assays in Circ_0003945‐knockdown or overexpressing HCC cells. Scale bar = 100 μm. (H and I) Migratory ability was measured by Transwell assay in Circ_0003945‐knockdown or overexpressing HCC cells. Scale bar = 50 μm. All in vitro experiments were performed in triplicate and t‐tests were used. (*p < 0.05; **p < 0.01; ***p < 0.001, ns, not significant)

To investigate the functional role of Circ_0003945 in HCC, the Circ_0003945 expression level in HCC cell lines was measured and showed that Circ_0003945 was highly expressed in most HCC cell lines compared with normal liver cell line (LO2; Figure 2A). Next, a specific shRNA targeting Circ_0003945 was designed and transfected into HCCLM3 and MHCC97H cells, and a Circ_0003945 overexpression vector was constructed and transfected into Li‐7 cells. Circ_0003945 expression was significantly decreased in HCCLM3 and MHCC97H cells (Figure 2B) and did not affect the level of its parental gene (UBAP2) at the mRNA and protein levels (Figure S3A,B). Dramatic upregulation of Circ_0003945 was found in overexpression groups (Figure 2C), with UBAP2 expression unaffected (Figure S3C‐3D).

Next, functional analysis showed that downregulation of Circ_0003945 attenuated viability of HCC cells (Figure 2D), whereas overexpression of Circ_0003945 increased viability of Li‐7 cells (Figure 2E). Wound‐healing assays implied that Circ_0003945 downregulation significantly decreased the migratory ability of HCC cells (Figure 2F), whereas Circ_0003945 overexpression revealed an opposite role (Figure 2G). Transwell assays showed a similar effect of Circ_0003945 in HCC cell lines (Figure 2H‐I).

### 3.3 Circ_0003945 acts as a miR‐34c‐5p sponge in HCC cells

**FIGURE 3:**
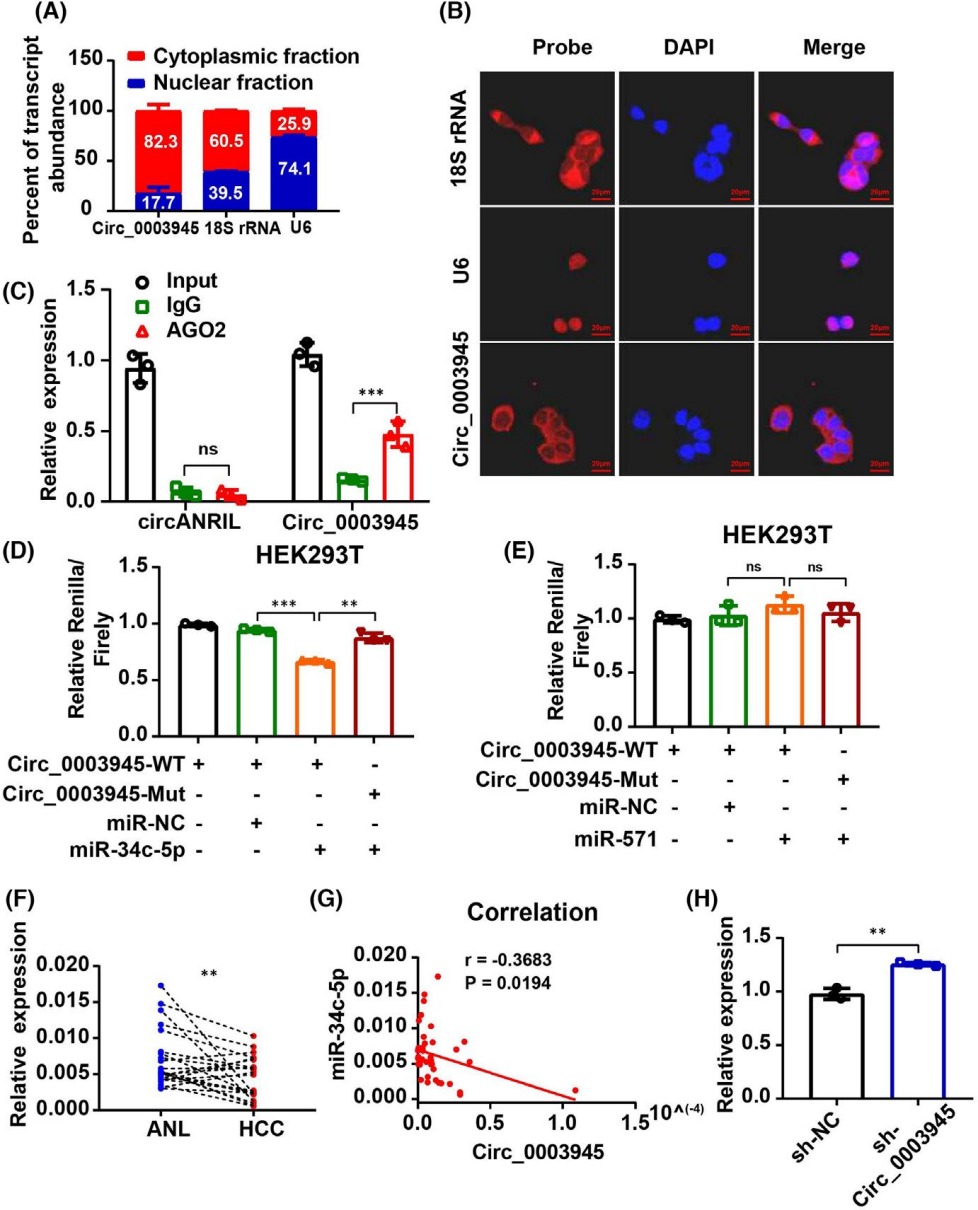
Circ_0003945 acts as miR‐34c‐5p sponge in HCC cells. (A) The location of Circ_0003945 was confirmed by subcellular fractionation. (B) FISH analysis was performed to observe the cellular location of Circ_0003945, 18S rRNA and U6 in MHCC97H cells. Circ_0003945, 18S rRNA and U6 probe were labelled with Cy3 (red) and nuclei were counterstained with DAPI (blue). Scale bar = 20 μm. (C) RIP experiment was performed to co‐immunoprecipitate AGO2 complex. (D and E) Relative luciferase activity in HEK‐293T cells co‐transfected with miR‐34c‐5p or miR‐571 and Circ_0003945‐WT or Circ_0003945‐Mut vector. (F) Expression level of miR‐34c‐5p was determined by qPCR and paired t‐test was used. (G) Correlation between miR‐34c‐5p and Circ_0003945 in tissue samples was evaluated using Spearman correlation analysis. (H) Expression level of miR‐34c‐5p in Circ_0003945‐knockdown HCC cells. All in vitro experiments were performed in triplicate and t‐test were used. (*p < 0.05; **p < 0.01; ns, not significant)

To explore the underlying mechanism of Circ_0003945 in HCC progression, subcellular fractionation analysis of Circ_0003945 was first performed to determine whether circRNAs play different roles depending on their localization in cells.^5^, ^20^ This analysis revealed that Circ_0003945 was mostly located in the cytoplasm of HCC cells (Figure 3A). Further FISH analysis also verified this (Figure 3B), implying that Circ_0003945 may act as a miRNA sponge to participate in HCC progression.

The regulatory effects of miRNAs on their target genes are dependent on RNA‐induced silencing complex (RISC), which contains the protein AGO2.^21^, ^22^ Therefore, RIP analysis was conducted using anti‐AGO2 antibody in HCC cells. We found that the anti‐AGO2 antibody significantly bound Circ_0003945, but not circANRIL (a circular RNA that does not bind AGO2 and was used as a negative control^23^, ^24^), indicating that Circ_0003945 might serve as a platform for AGO2 and miRNAs (Figure 3C).

Subsequently, the putative miRNA targets were predicted using three publicly available prediction tools (CircInteractome, circBank and miRanda). Two miRNAs (hsa‐miR‐34c‐5p [miR‐34c‐5p)] and hsa‐miR‐571 [miR‐571]) were selected as possible targets of Circ_0003945 for their high comprehensive score (Figure S4A; Table S2). A further dual‐luciferase reporter assay was performed to investigate whether miR‐34c‐5p or miR‐571 targets Circ_0003945 (Figure S4B). MiR‐34c‐5p mimics or miR‐571 mimics were co‐transfected with the luciferase reporters (which contained wild‐type or mutant miR‐34c‐5p/miR‐571 target sequences of Circ_0003945) into HEK‐293T cells. Compared with the negative control miRNA (miR‐NC), miR‐34c‐5p significantly decreased luciferase reporter activity in the cells compared with the wild‐type Circ_0003945 sequence, but not in mutant binding groups (Figure 3D). However, no significant effect was found in a similar experimental system where cells were transfected with miR‐571 mimics (Figure 3E). Therefore, Circ_0003945 might be a miR‐34c‐5p sponge.

Next, the association between Circ_0003945 and miR‐34c‐5p was addressed. The miR‐34c‐5p was expressed at a lower level in HCC tissue than in adjacent normal tissue (Figure 3F), and a negative correlation was found between Circ_0003945 and miR‐34c‐5p in tissue samples (Figure 3G). Further, knockdown of Circ_0003945 upregulated miR‐34c‐5p expression level (Figure 3H).

### 3.4 Transfection of Circ_0003945 antagonizes the inhibitory effects of miR‐34c‐5p on HCC progression *in vitro*

**FIGURE 4:**
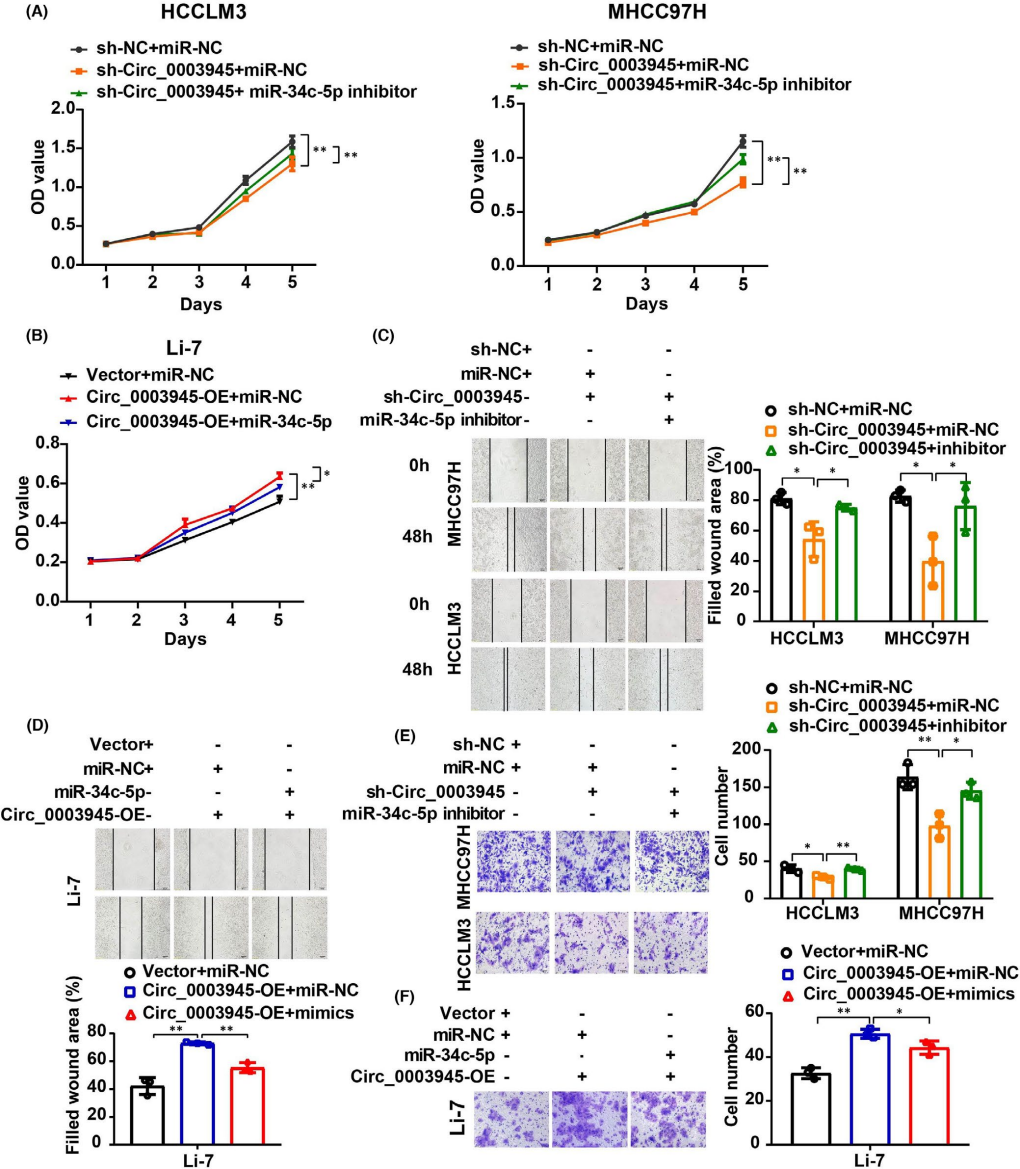
Enhanced expression of Circ_0003945 attenuates miR‐34c‐5p‐mediated role of HCC progression *in vitro*. (A and B) Viability of HCC cells with changing expression of Circ_0003945 or miR‐34c‐5p or both via CCK‐8 assay. (C and D) Migratory ability of HCC cells with altering expression of Circ_0003945 and miR‐34c‐5p assessed by wound‐healing assay. Scale bar = 100 μm. (E and F) The migratory ability of HCC cells with changing expression of Circ_0003945 and miR‐34c‐5p was assessed by Transwell assay. Scale bar = 50 μm. All in vitro experiments were performed in triplicate and t‐tests were used. (*p < 0.05; **p < 0.01)

Next, functional analysis was carried out to evaluate the effect of Circ_0003945 sponging miR‐34c‐5p. Results showed that inhibiting miR‐34c‐5p expression reversed the reduced cell viability induced by Circ_0003945 knockdown (Figure S4C; Figure 4A), and the miR‐34c‐5p mimics attenuated the promotional effects of Circ_0003945 overexpression on proliferation (Figure S4D; Figure 4B). By analogy, suppressing miR‐34c‐5p expression enhanced the role of Circ_0003945 in maintaining the migratory ability of HCC cells (Figure 4C). Meanwhile, miR‐34c‐5p mimics dramatically inhibited the migration of HCC cells induced by Circ_0003945 overexpression (Figure 4D). A similar effect was observed in Transwell assays (Figure 4E‐F). These results collectively illustrate that Circ_0003945 is necessary to maintain HCC cell progression in part by absorbing miR‐34c‐5p.

### 3.5 Circ_0003945 contributes to HCC progression by promoting LGR4 expression by sponging miR‐34c‐5p

The miRNAs have been reported to regulate their downstream target genes by recognizing the guide sequence. The downstream targets of miR‐34c‐5p were predicted according to the TargetScan and miRDB databases. Based on the comprehensive score, immunoglobulin superfamily member 1 (IGSF1), CUE domain‐containing protein 1 (CUEDC1), cell death‐inducing p53 target 1 (CDIP1), small G protein signalling modulator 2 (SGSM2), and leucine‐rich repeat‐containing G protein‐coupled receptor 4 (LGR4) were finally selected for further verification (Table S3). The qPCR results showed that only LGR4 expression was dramatically decreased by transfection of miR‐34c‐5p mimics compared with miR‐NC, and LGR4 was upregulated by suppressing miR‐34c‐5p expression (Figure S5A). Western blot analysis also confirmed this (Figure S5B). Further, luciferase reporters were constructed to verify this interaction (Figure S5C). Luciferase assay showed that transfection of miR‐34c‐5p mimics dramatically decreased the activity of the luciferase reporter carrying the wild‐type LGR4 3’ untranslated region (3’‐UTR) compared with the miR‐NC group. In contrast, the mutated luciferase reporter showed no significant change with miR‐34c‐5p overexpression (Figure S5D). Additionally, LGR4 was expressed at remarkably high level in HCC tissue compared with adjacent normal liver specimens (Figure S5E). A negative correlation was also found between the level of miR‐34c‐5p and LGR4 expression in tissue samples (Figure S5F).

**FIGURE 5:**
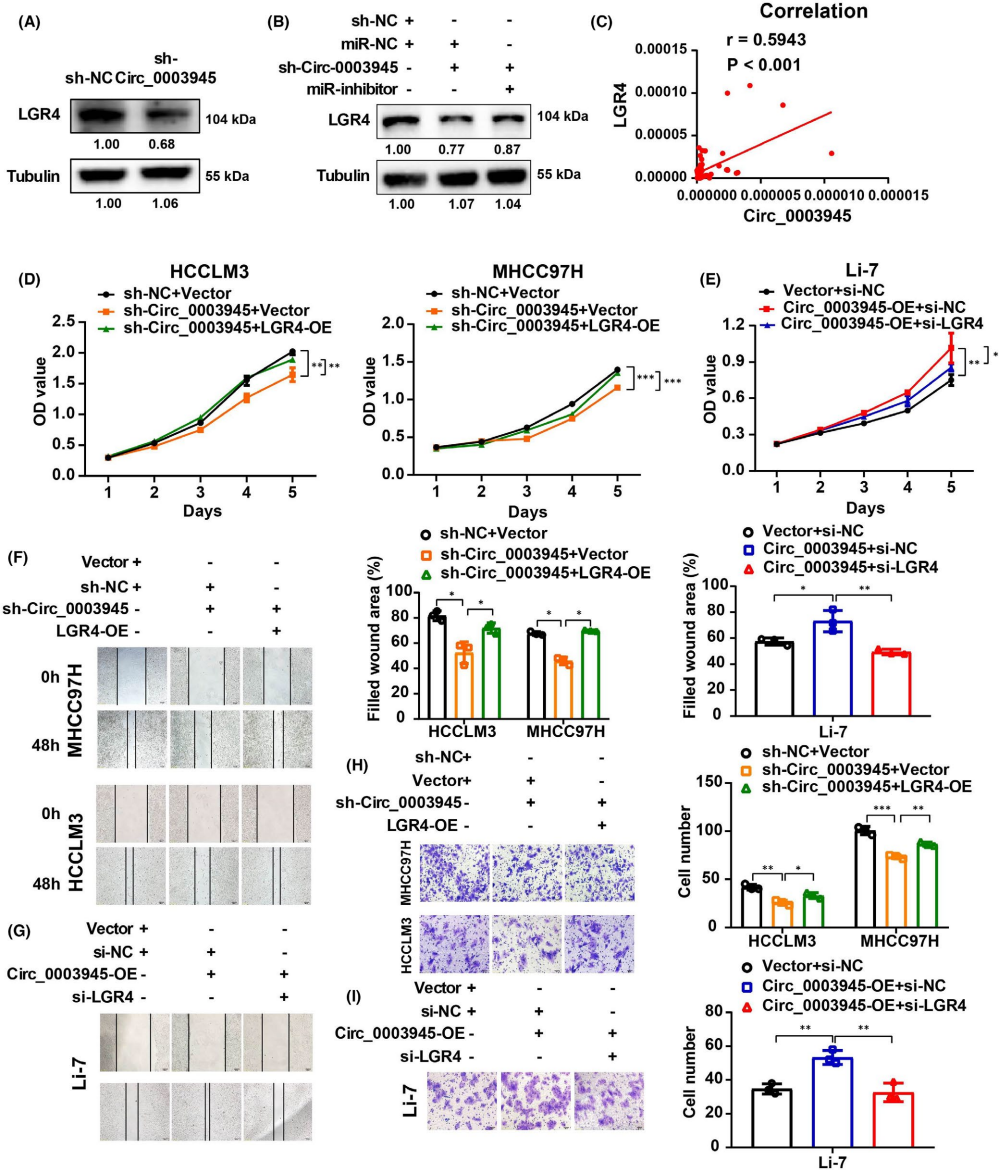
Circ_0003945 contributes to HCC progression by regulating LGR4. (A) Western blot analysis of LGR4 in Circ_0003945‐knockdown HCC cells. (B) Level of LGR4 in HCC cells with Circ_0003945‐knockdown or miR‐34c‐5p alteration. (C) The correlation between LGR4 and Circ_0003945 expression; Spearman's rank correlation test was used. (D and E) Proliferation assay of HCC cells with silencing or overexpressing Circ_0003945 and LGR4. (F‐I) Migration of HCC cells after silencing or overexpressing Circ_0003945 and LGR4. For F and G, scale bar = 100 μm, for H and I, scale bar = 50 μm. Functional experiments were performed in triplicate and t‐tests were used. (*p < 0.05; **p < 0.01; ***p < 0.001)

As Circ_0003945 may function as a miR‐34c‐5p sponge to regulate LGR4 expression, the relationship between Circ_000345 and LGR4 was also addressed. Western blot analysis revealed that LGR4 expression significantly decreased with decreasing level of Circ_0003945 (Figure 5A and Figure S5G), and the suppressed LGR4 level in Circ_00039450‐knockdown HCC cells was promoted by miR‐34c‐5p inhibition (Figure 5B and Figure S5H). Clinically, we also found that Circ_0003945 level was positively correlated with LGR4 expression (Figure 5C).

We further explored the biological role of LGR4 induced by Circ_0003945. Functional analysis showed that repressed proliferation of Circ_0003945‐knockdown HCC cells was rescued by LGR4 overexpression (Figure 5D). Meanwhile, enhanced proliferation of Circ_0003945‐overexpressing HCC cells was suppressed by suppressing LGR4 expression (Figure 5E). The migration assay revealed similar results (Figure 5F‐5I).

### 3.6 Circ_0003945/miR‐34c‐5p/LGR4 promotes HCC progression via affecting β‐catenin pathway

**FIGURE 6:**
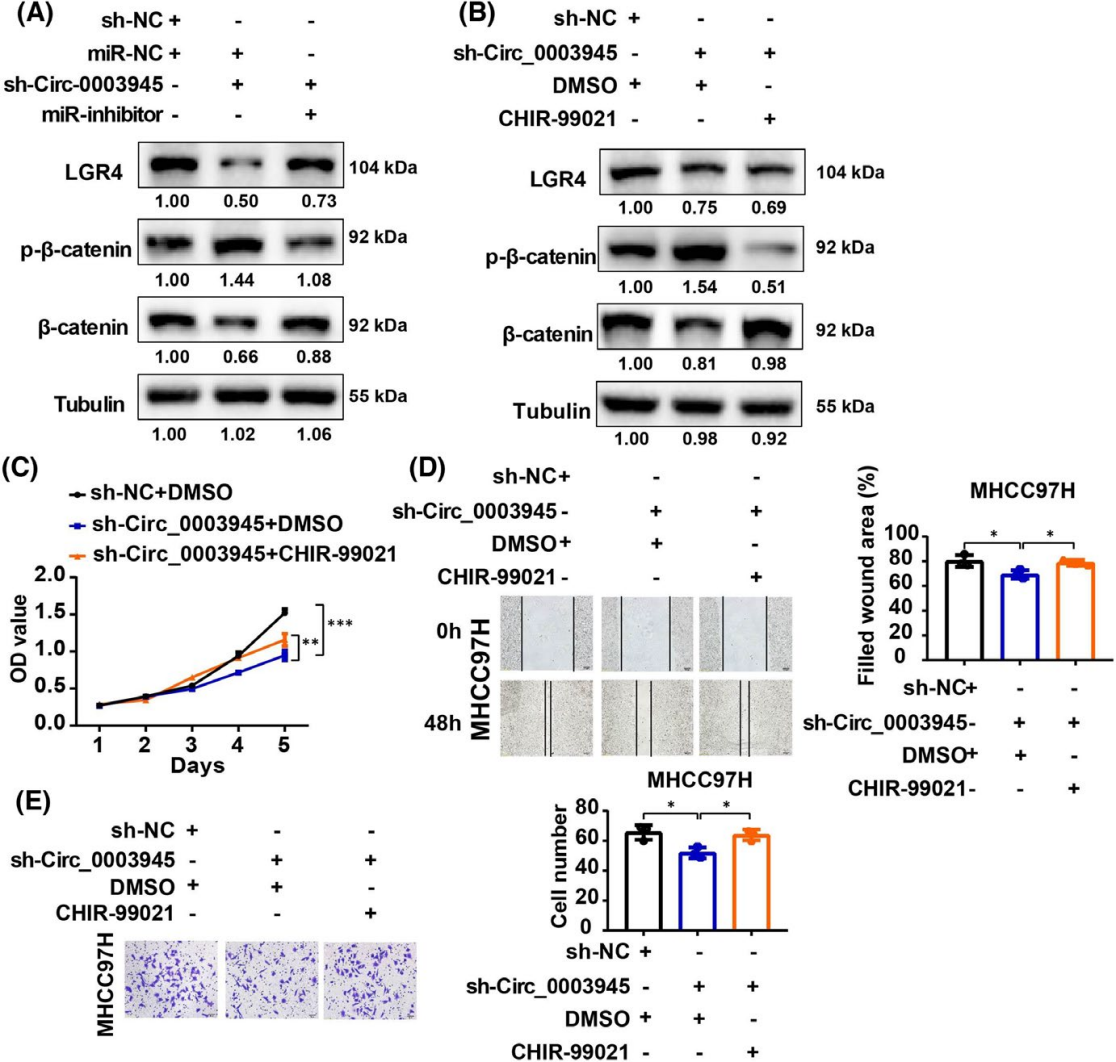
β‐Catenin pathway was finally activated by Circ_0003945/miR‐34c‐5p/LGR4 to promote HCC progression. (A) Western blot analysis of the β‐catenin pathway induced by the Circ_0003945/miR‐34c‐5p axis in HCC cells. (B) The effect of β‐catenin activator on Circ_0003945‐knockdown HCC cells. (C) Measurement of proliferation of HCC cells treated with β‐catenin activator after silencing of Circ_0003945 in HCC cells. (D‐E) Migratory ability of decreasing‐Circ_0003945‐HCC cells after treatment with β‐catenin activator. For figure D, scale bar = 100 μm. For figure E, scar bar = 50 μm. All experiments were performed in triplicate and t‐tests were used. (*p < 0.05; **p < 0.01; ***p < 0.001)

To identify the signalling pathway underlying the effects of the Circ_0003945/miR‐34c‐5p/LGR4 axis on HCC cells, KEGG analysis (Entry:map 04310) and previous studies showed that LGR4 is the key gene in the Wnt/β‐catenin pathway.^25^, ^26^ Therefore, we investigated whether β‐catenin pathway was influenced by the Circ_0003945/miR‐34c‐5p/LGR4 axis. It revealed that stable knockdown of Circ_0003945 contribute to the phosphorylation of β‐catenin and miR‐34c‐5p reversed this effect (Figure 6A and Figure S6A), which may induce accumulation of β‐catenin. Nuclear and cytoplasmic protein assays also confirmed that the accumulation of β‐catenin in the nucleus in overexpressing‐Circ_0003945 HCC cells (Figure S6B). The representative downstream target genes of the β‐catenin pathway (c‐Myc and Cyclin D1) were attenuated by silencing Circ_0003945 (Figure S6C,D) and miR‐34c‐5p also participated in this process.

To further determine whether the β‐catenin pathway is regulated by Circ_0003945, CHIR‐99021^27^ (an activator of Wnt/β‐catenin) was used. We found that CHIR‐99021 suppressed the phosphorylation of β‐catenin in Circ_0003945‐knockdown MHCC97H cells (Figure 6B; Figure S6E). In addition, CCK‐8 assay showed that CHIR‐99021 reversed the reduced cell viability of Circ_0003945 knockdown (Figure 6C). Transwell assays also indicated a similar role of CHIR‐99021 in migration and invasion of HCC cells (Figure 6D,E). Moreover, β‐catenin was knockdown in Circ_0003945‐overexpressing HCC cells, and we found that Circ_0003945 enhanced cell proliferation and migration, while intervening β‐catenin could inhibit the enhanced role of Circ_0003945 (Figure S7A‐C).

### 3.7 Circ_0003945 promotes the growth of HCC tumours *in vivo*

**FIGURE 7:**
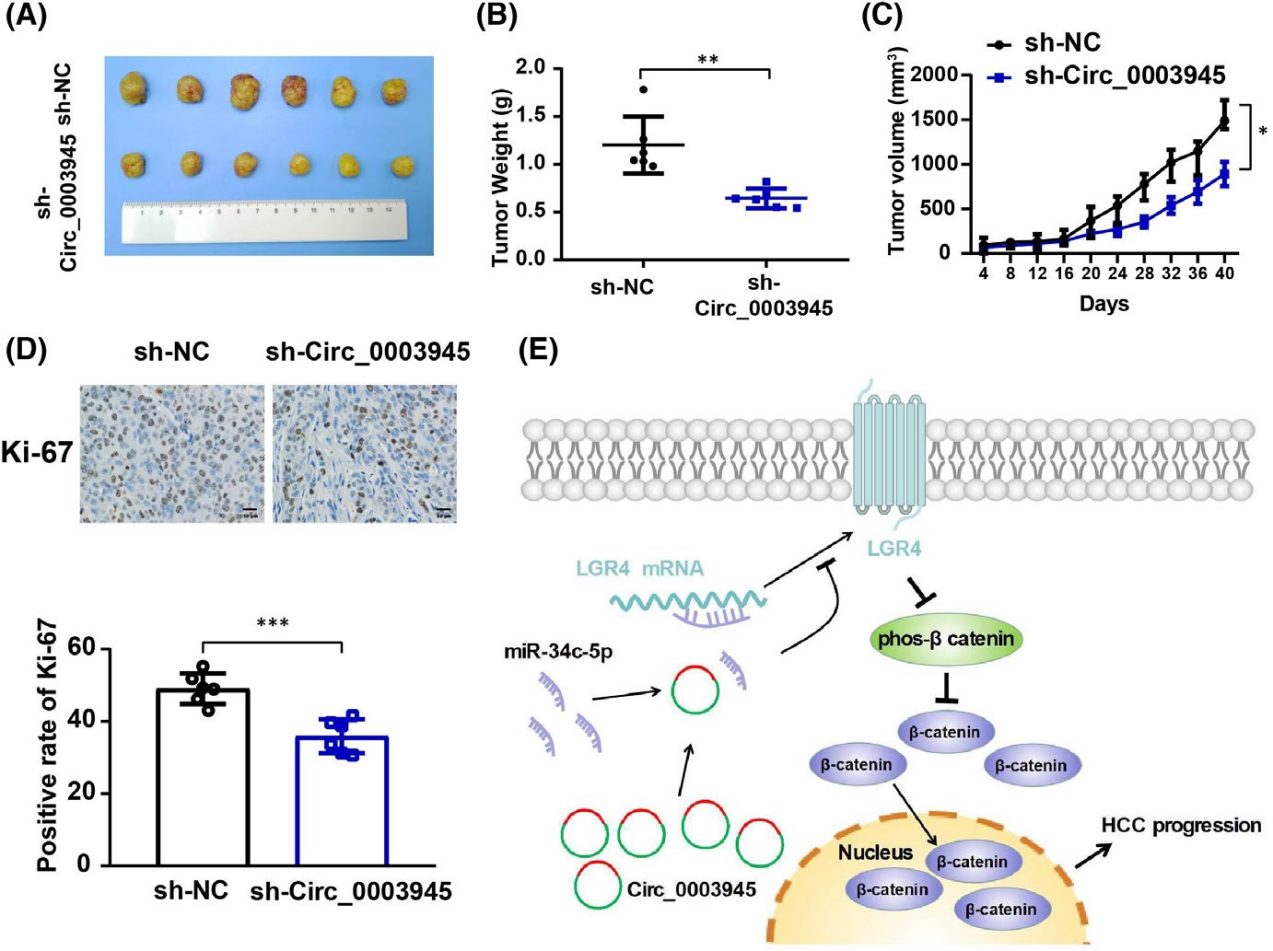
Circ_0003945 promotes the growth and metastasis of HCC cells *in vivo*. (A) Tumours were dissected from nude mice subcutaneously injected with Circ_0003945‐knockdown or control MHCC97H cells. (B and C) Weight and volume of subcutaneous xenograft tumours isolated from nude mice. (D) Representative images of immunohistochemical analysis to detect Ki‐67. Scar bar = 50 μm. (E) Schematic diagram indicating the underlying mechanism by which Circ_0003945 enhances HCC progression via the miR‐34c‐5p/LGR4 axis. (*p < 0.05; **p < 0.01; ***p < 0.001)

To validate the phenotype of Circ_0003945 in HCC progression *in vivo*, we subcutaneously injected nude mice with Circ_0003945‐knockdown HCCLM3 cells. Circ_0003945 knockdown significantly decreased tumour volume and tumour weight in nude mice (Figure 7A‐C). Further, immunohistochemical analysis of tumours generated from HCC cells showed that the level of Ki‐67 expression (an indicator of cell proliferation) was markedly decreased in Circ_0003945‐knockdown groups (Figure 7D). In conclusion, Circ_0003945 acts as a miR‐34c‐5p sponge to upregulate LGR4, activating the β‐catenin axis and promoting HCC progression (Figure 7E).

## 4 DISCUSSION

Although accumulating research has revealed that circRNAs clearly show abnormal expression related to tumour growth and progression in HCC,^17^, ^28^, ^29^, ^30^ the function of numerous circRNAs in HCC remains elusive and needs to be further investigated. In the current study, the expression of Circ_0003945, originating from exon 11 and 12 of UBAP2, was significantly upregulated in HCC tissue and plasma. Further analysis indicated that higher expression of Circ_0003945 was correlated with tumour size and tumour stage in HCC tissue. Functionally, Circ_0003945 affected a series of properties of HCC cells, including tumour cell proliferation and migration. Mechanistically, Circ_0003945 acted as a miR‐34c‐5p sponge to upregulate LGR4 expression, contributing to HCC progression by regulating β‐catenin pathway activity. Thus, our data provide a new perspective on tumour‐promoting roles of Circ_0003945 in HCC, which might be a promising therapeutic target for HCC treatment.

Abnormal expression of circUBAP2 has been found in several cancer types, and a total of 90 circRNAs originating from UBAP2 have been identified, whereas specific back‐splicing sites of these circRNAs still need to be further elucidated, and they might be derived from different exons. Meanwhile, few studies have explored the downstream signalling pathway induced by the circRNA/miRNA/mRNA axis. Here, we demonstrated that Circ_0003945, a circUBAP2, plays the key role in HCC tumorigenesis and progression. Our study provides new evidence that Circ_0003945 acts as a miR‐34c‐5p sponge and thus further protects β‐catenin from phosphorylation, which may cause accumulation of β‐catenin and trigger a series of gene transcription events. Taken together, our data comprehensively confirm that Circ_0003945/miR‐34c‐5p/LGR4/β‐catenin pathway exerts its tumour‐promoting role in HCC progression. Interestingly, Circ_0003945 could be also sponged with miR‐194‐3p and upregulate MMP9 (one of the downstream target gene of β‐catenin^31^), finally facilitating HCC progression.^32^ Thus, Cir_0003945/β‐catenin axis might be a potential therapeutic target for HCC treatment.

Additionally, circRNAs are considered potential diagnostic and prognostic biomarkers.^15^, ^33^, ^34^, ^35^ A plasma circular RNA panel (hsa_circ_0000976, hsa_circ_0007750 and hsa_circ_0139897) was constructed to diagnose hepatitis B–virus‐related HCC.^36^ Although a previous study found that higher circUBAP2 expression can predict poor outcome for cervical cancer patients,^37^ there is no data exploring the expression level of free circUBAP2 in the circulation. In this study, elevated expression of Circ_0003945 was identified in HCC tissue and correlated with HCC stage. Furthermore, a high level of plasma circulating Circ_0003945 was confirmed in HCC patients compared with that in non‐HCC groups. These findings imply that Circ_0003945 might be a novel biomarker for HCC diagnosis and prognosis, as well as surveillance for treatment. However, a large‐scale, prospective, multicentre study is still needed for further confirmation. It is noteworthy that we just focus on free circulating Circ_0003945 level in this study, other sources for this circular RNA in the body should also be confirmed, such as whether it exists in exosomes. Accumulating evidence proposed that circRNAs could be enriched in exosomes and own potential diagnosis or prognostic value for HCC.^35^, ^38^, ^39^, ^40^ Therefore, the clinical performance of exosomal Circ_0003945 needs to be elucidated in the future.

Although the tumour‐promoting effects of Circ_0003945 were verified to be as a miR‐34c‐5p sponge, other potential underlying mechanisms, such as interaction with proteins or translation into polypeptides, should also be considered. Considering that exosomal circular RNAs also play contribute to tumour progression,^41^, ^42^, ^43^ the physiological function of exosomal Circ_0003945 require further exploration.

In summary, we propose that Circ_0003945 is significantly upregulated in HCC cells and is related to HCC progression. Functionally and mechanistically, Circ_0003945 might directly target miR‐34c‐5p to release its inhibition of LGR4 expression, finally activating the β‐catenin pathway, and thus, enhancing malignant properties of HCC cell. Therefore, Circ_0003945 might serve as a novel biomarker and therapeutic target for anti‐HCC therapy.

## 5 ETHICS STATEMENT

The present study was approved by the Medical Ethics Committee of Zhongshan Hospital, Fudan University. All participants in this study provided written informed consent in accordance with the Declaration of Helsinki.

## CONFLICT OF INTEREST

The authors declare no competing interests.

## AUTHOR CONTRIBUTIONS

**Lihua Lyu:** Data curation (lead); Formal analysis (lead); Methodology (lead); Software (equal); Validation (lead); Visualization (lead); Writing – original draft (lead). **Chunyan Zhang:** Data curation (equal); Funding acquisition (equal); Supervision (equal). **Wenjing Yang:** Conceptualization (equal); Data curation (equal); Formal analysis (equal); Supervision (equal); Writing – original draft (equal). **Anli Jin:** Data curation (equal); Formal analysis (equal); Methodology (equal); Resources (equal); Software (equal). **Jie Zhu:** Data curation (equal); Formal analysis (equal); Resources (equal); Software (equal). **Hao Wang:** Data curation (equal); Formal analysis (equal); Software (equal); Writing – original draft (equal). **Te Liu:** Conceptualization (equal); Visualization (equal); Writing – original draft (equal); Writing – review & editing (equal). **Beili Wang:** Conceptualization (equal); Funding acquisition (equal); Project administration (equal); Visualization (equal); Writing – review & editing (equal). **Jianwen Cheng:** Data curation (equal); Methodology (equal); Resources (equal); Writing – original draft (equal). **Xinrong Yang:** Funding acquisition (equal); Supervision (equal); Validation (equal); Writing – review & editing (equal). **Wei Guo:** Conceptualization (lead); Funding acquisition (lead); Writing – review & editing (equal).

## Supporting information

Supplementary Material

Supplementary Material

## Data Availability

The datasets analysed for this study can be found in the Gene Expression Omnibus (GSE78520, GSE94508 and GSE97332).
