# Supplementary material for: Hsa_circ_0003945 promotes progression of hepatocellular carcinoma by mediating miR‐34c‐5p/LGR4/β‐catenin axis activity
Source: J Cell Mol Med. 2022 Feb 16;26(8):2218–29. doi: 10.1111/jcmm.17243 (PMC8995453; doi:10.1111/jcmm.17243)
Supplement: Supplementary file 1 — Supplementary Material [file JCMM-26-2218-s002.doc]

**Hsa_circ_0003945 promotes progression of hepatocellular carcinoma by mediating miR-34c-5p/LGR4/β-catenin axis activity**

Li-Hua Lyu^1#^, Chun-yan Zhang^1, 4#^, Wen-Jing Yang^1^, An-Li Jin^1^, Jie Zhu^1^, Hao Wang^1^, Te Liu^5^, Bei-Li Wang^1, 3^, Jian-Wen Cheng^2, 3^, Xin-Rong Yang^2, 3^*, Wei Guo^1, 3, 4, 6^*

^1^Department of Laboratory Medicine, Zhongshan Hospital, Fudan University, Shanghai, China.

^2^Department of Liver Surgery & Transplantation, Liver Cancer Institute, Zhongshan Hospital, Fudan University; Key Laboratory of Carcinogenesis and Cancer Invasion, Ministry of Education, Shanghai, China.

^3^Cancer center，Zhong Shan Hospital，Fudan University, Shanghai, China.

^4^Department of Laboratory Medicine, Xiamen Branch，Zhongshan Hospital, Fudan University, Xiamen, China.

^5^Shanghai Geriatric Institute of Chinese Medicine, Shanghai University of Traditional Chinese Medicine, Shanghai, China.

^6^Department of Laboratory Medicine, Wusong Branch，Zhongshan Hospital, Fudan University, Shanghai, China.

**Supplementary Figures**

**
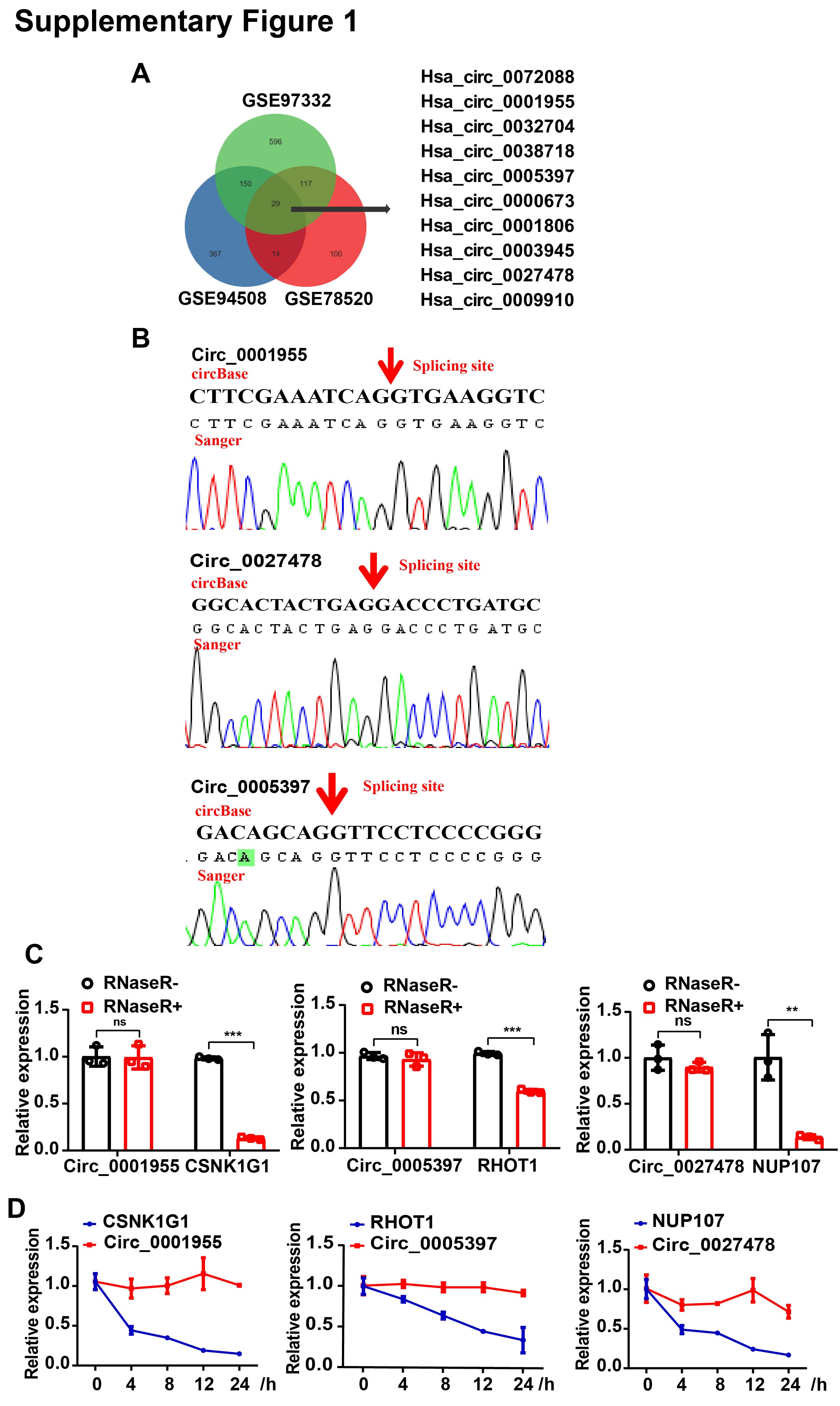
**

**Supplementary Figure 1.** **Characteristics of dysregulated circRNAs. (A)** Venn diagram showing the intersection of dysregulated circRNAs in GEO datasets and ten circRNAs selected for the next study. **(B)** Sanger sequencing was performed to detect the back-splicing sites (red arrows) of Circ_0001955, Circ_0027478, and Circ_0005397, respectively. **(C and D)** The qPCR analysis of candidate circRNAs and their associated mRNAs in HCC cells treated with or without RNase R or actinomycin D, respectively. Experiments were performed in triplicate and t tests were used. (**P < 0.01; ***P < 0.001; ns, not significant).


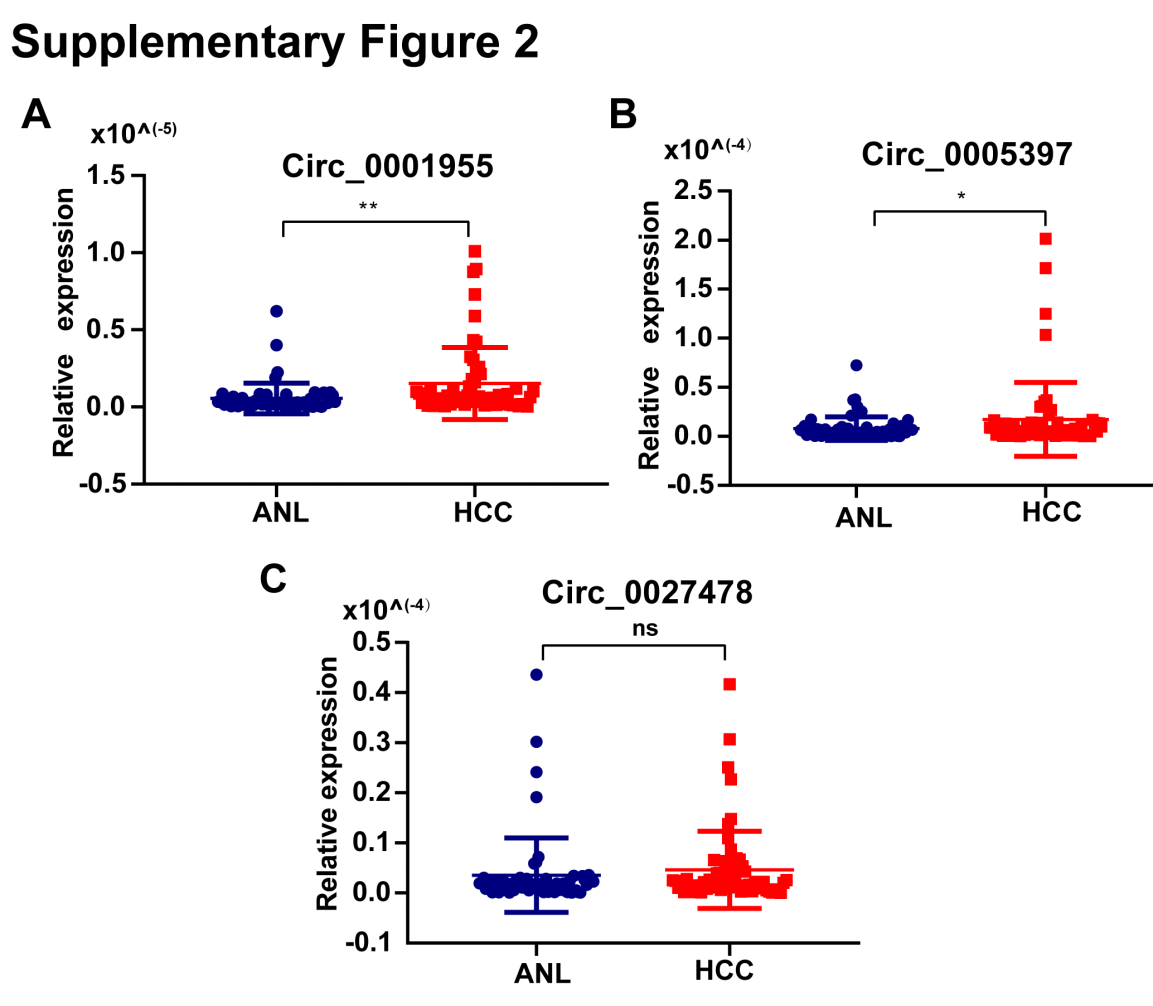


**Supplementary Figure 2.** **Expression of candidate circRNAs in HCC tissue. (A-C)** Expression levels of candidate circRNAs were analyzed by qPCR in HCC and ANL tissues; paired t test was used. (*P < 0.05; **P < 0.01; ns, not significant).


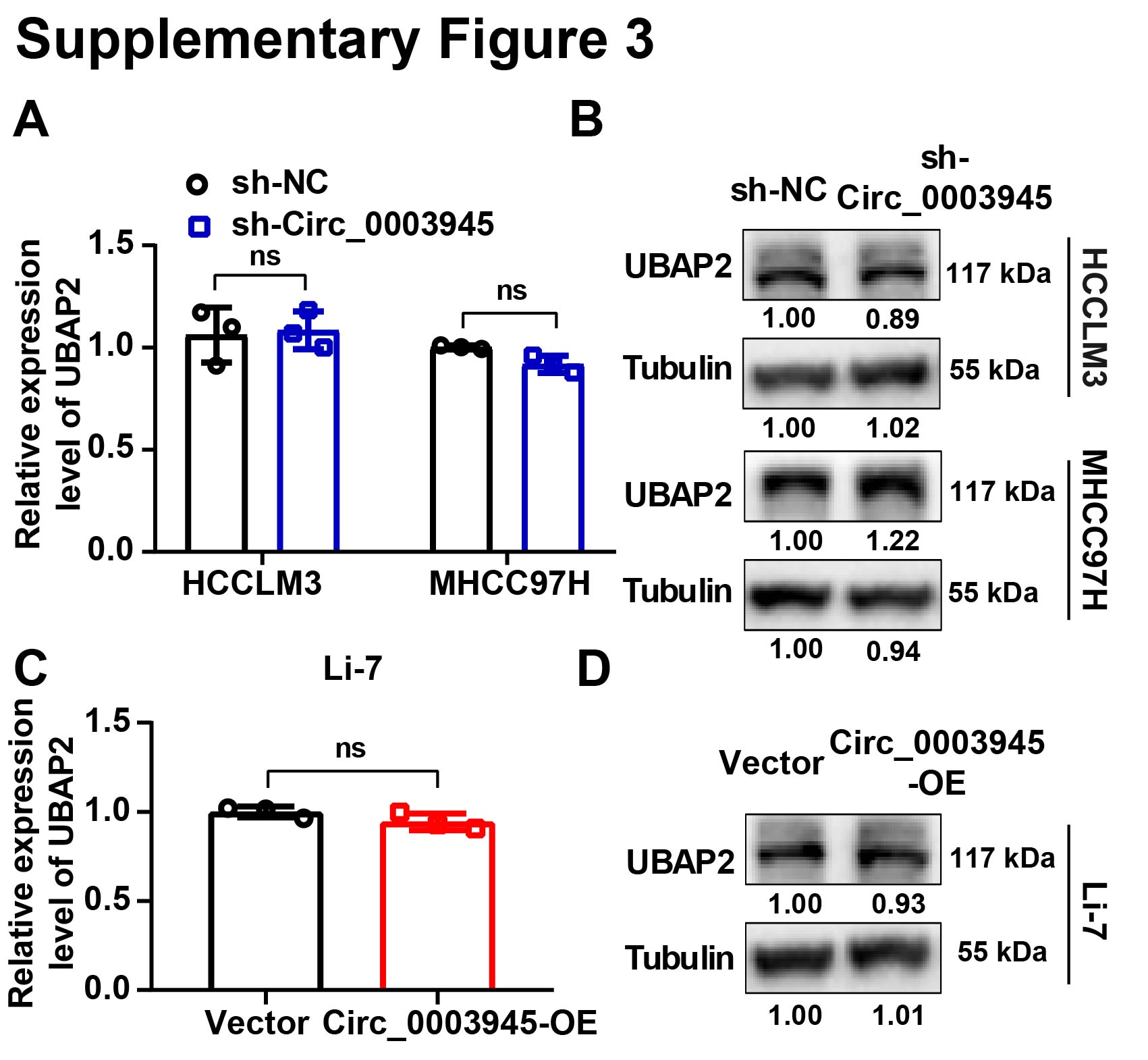


**Supplementary Figure 3. Effects of Circ_0003945 on parental gene in HCC cells. (A and B)** Effect of Circ_0003945 knockdown on its parental gene. **(C and D)** Effect of overexpressing Circ_0003945 on its parental gene. Experiments were performed in triplicate and t tests were used. (ns, not significant).


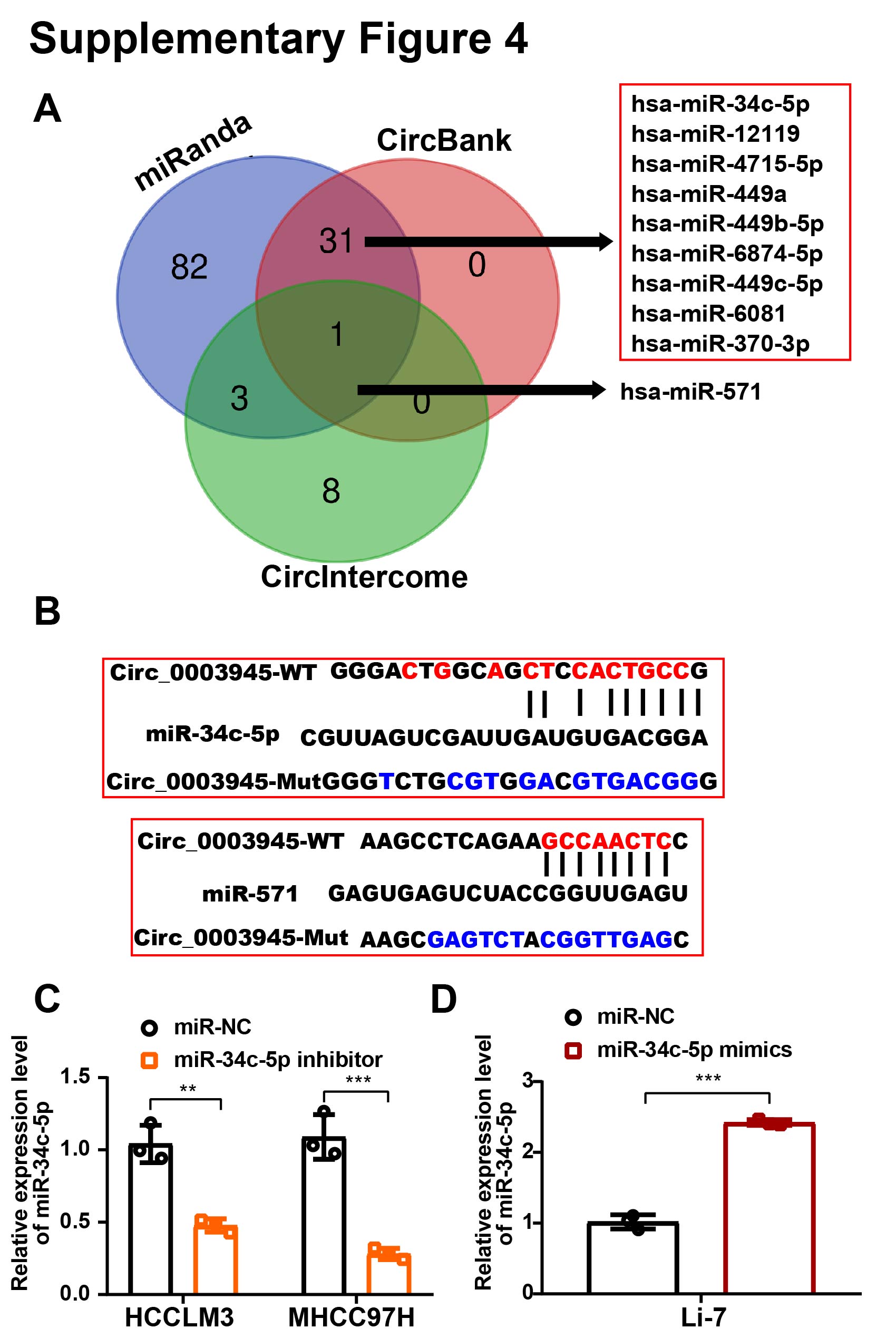


**Supplementary Figure 4. Potential miRNA targets binding to Circ_0003945. (A)** Potential miRNA targets binding to Circ_0003945 predicted by online database. **(B)** Schematic representation of binding sites of miR-34c-5p and miR-571 on Circ_0003945 are indicated by red font. Blue font indicates the mutated sequences of Circ_0003945. **(C and D)** The transfection efficiency of miR-34c-5p. Experiments were performed in triplicate and t tests were used. (**P < 0.01; ***P < 0.001; ns, not significant).

**
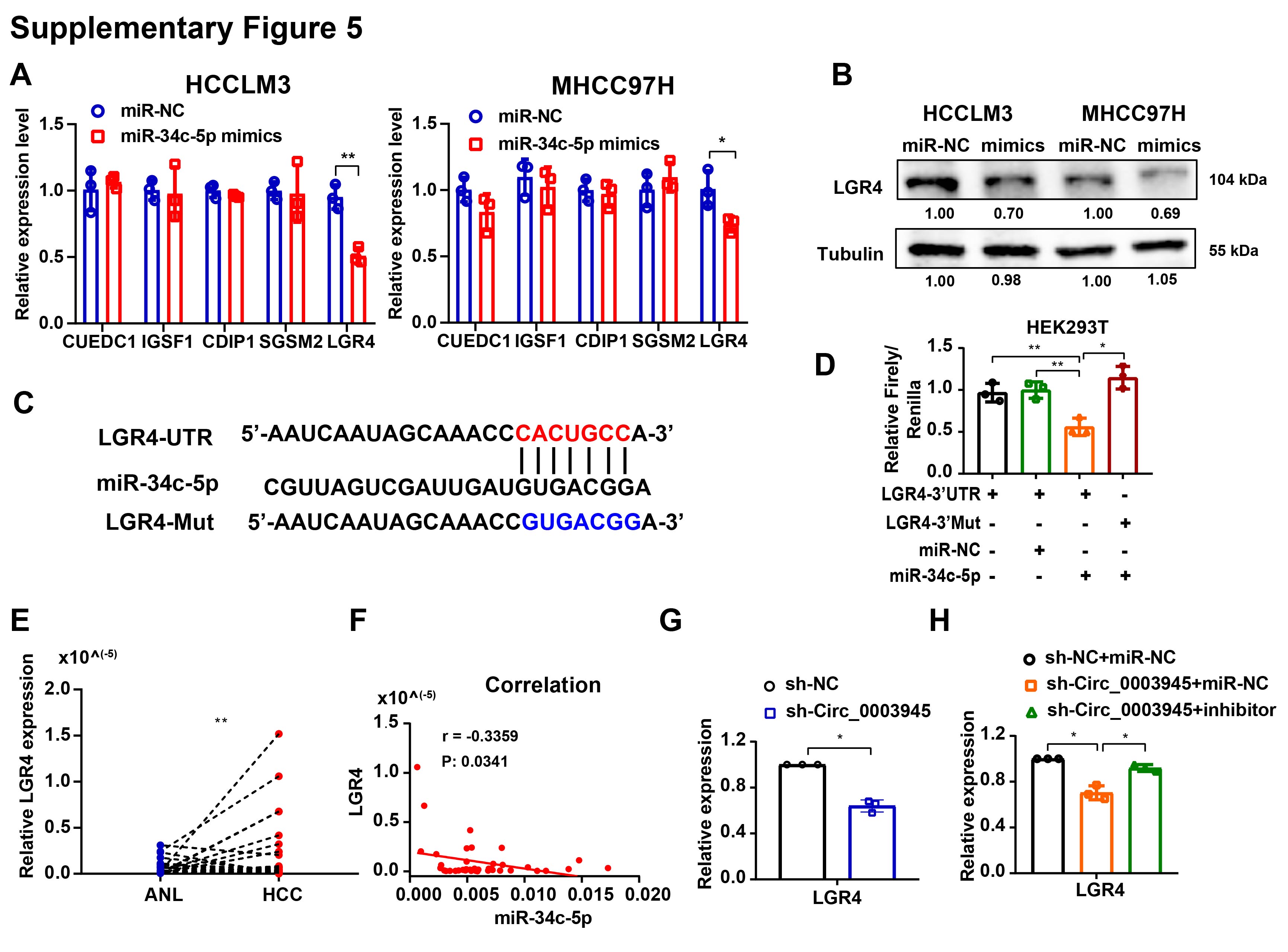
**

**Supplementary Figure 5. LGR4 is the downstream target of miR-34c-5p. (A)** The expression level of potential downstream targets of miR-34c-5p in HCC cells after upregulation of miR-34c-5p. **(B)** Western blot analysis of LGR4 in HCC cells transfected with miR-34c-5p mimics. **(C)** Schematic representation of binding sites of miR-34c-5p on LGR4 (red font) and mutated sequences of LGR4 (blue font). **(D)** Dual-luciferase reporter assay was performed to determine whether LGR4 is the target of miR-34c-5p. **(E)** Expression of LGR4 was measured by qPCR; paired t test was used. **(F)** Correlation between LGR4 and miR-34c-5p expression was analyzed by Pearson’s test. **(G)** The quantitative results of western blot analysis of LGR4 in Circ_0003945-knockdown HCC cells. **(H)** The quantitative results of western blot analysis of LGR4 in HCC cells with Circ_0003945-knockdown or miR-34c-5p. Experiments were performed in triplicate. (*P < 0.05; **P < 0.01).


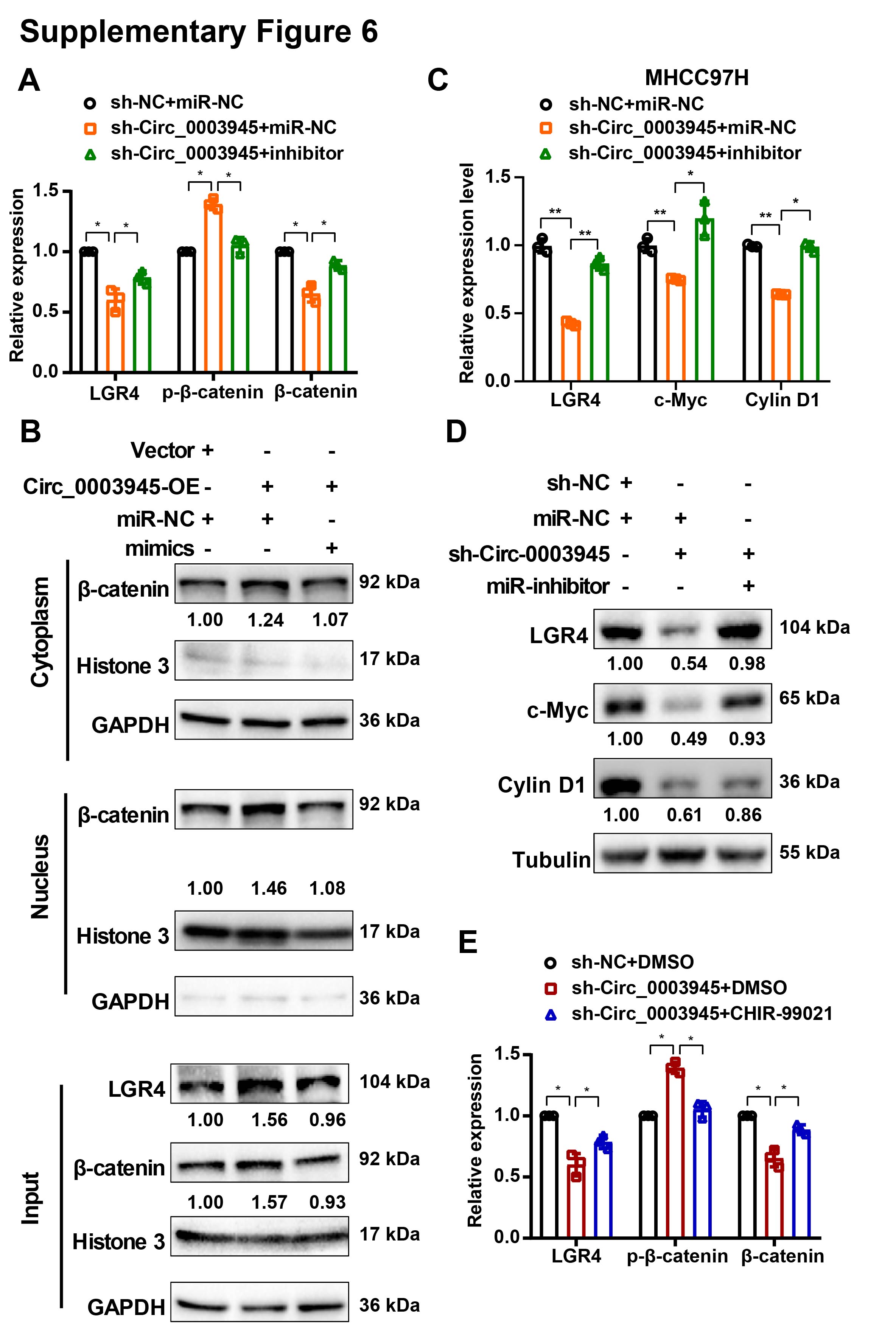


**Supplementary Figure 6. The Circ_0003945/miR-34c-5p/LGR4 axis ultimately promotes the β-catenin pathway in HCC cells. (A)** The quantitative results of western blot analysis of the β-catenin pathway induced by the Circ_0003945/miR-34c-5p axis in HCC cells. **(B)** Nuclear and cytoplasmic protein analysis of the accumulation of β-catenin in nuclei. **(C-D)** The known downstream targets of β-catenin measured by qPCR and western blotting. **(E)** The quantitative results of β-catenin pathway induced by CHIR-99021. (*P < 0.05; **P < 0.01).

**
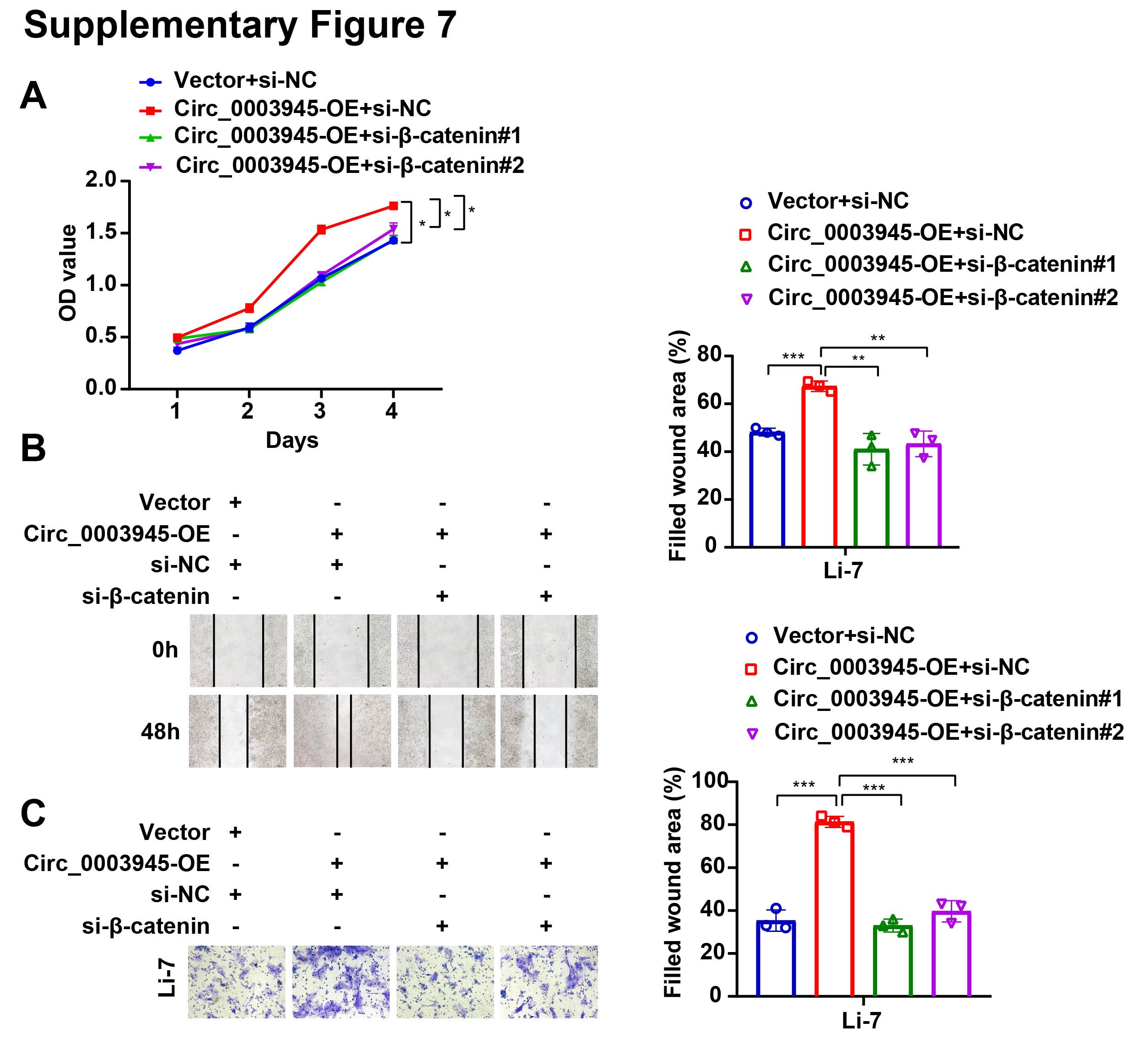
**

**Supplementary Figure 7. The Circ_0003945 influenced proliferation and migration of HCC cellsp via β-catenin pathway. (A)** Whether β-catenin was involved with Circ_0003945 on the proliferation of HCC cells was monitored via CCK-8 assays. **(B and C)** Whether β-catenin was involved with Circ_0003945 on the migration of HCC cells were monitored via would healing (Scale bar = 100 μm) and transwell assays (Scale bar = 50 μm). Experiments were performed in triplicate and t tests were used. (*P<0.05; **P < 0.01; ***P < 0.001).

**Supplementary Tables**

**Supplementary Table 1. The information of 10 up-regulated candidate circRNAs screened form databases**

| **circBase ID** | **Host gene Symbol** | **Genomic Position** | **Length** | **logFC** |
| --- | --- | --- | --- | --- |
| Hsa_circ_0072088 | ZFR | chr5: 32379220-32388780 strand: - | 693 | 4.6531421 |
| Hsa_circ_0001955 | CSNK1G1 | chr15: 64495280-64508912 strand: - | 815 | 3.8742393 |
| Hsa_circ_0032704 | TTLL5 | chr14: 76173360-76187046 strand: + | 457 | 3.0933442 |
| Hsa_circ_0038718 | IL4R | chr16: 27351506-27353580 strand: + | 227 | 2.312638 |
| Hsa_circ_0005397 | RHOT1 | chr17: 30500849-30503232 strand: + | 233 | 3.766187 |
| Hsa_circ_0000673 | RSL1D1 | chr16: 11940357-11940700 strand: - | 251 | 2.386035 |
| Hsa_circ_0001806 | CSPP1 | chr8: 68018139-68028357 strand: + | 432 | 2.3077778 |
| Hsa_circ_0003945 | UBAP2 | chr9: 33953282-33956144 strand: - | 258 | 2.5416102 |
| Hsa_circ_0027478 | NUP107 | chr12: 69109406-69125499 strand: + | 1029 | 2.4430321 |
| Hsa_circ_0009910 | MFN2 | chr1: 12049221-12052747 strand: + | 315 | 1.7049311 |

ZFR, zinc finger RNA binding protein; CSNK1G1, casein kinase 1 gamma 1; TTLL5, tubulin tyrosine ligase like 5; IL4R, interleukin 4 receptor; RHOT1, ras homolog family member T1; RSL1D1, ribosomal L1 domain containing 1; CSPP1, centrosome and spindle pole associated protein 1; UBAP2, ubiquitin associated protein 2; NUP107, nucleoporin 107; MFN2, mitofusin 2; logFC, the maximum log fold change among three databases.

**Supplementary Table 2. The miRNA predictions binding to circ_0003945 in database**

| **miRNA ID** | **Total Score** | **Total Energy** | **Max Score** | **Max Energy** | **MiRLen** | **CircLen** | **Positions** | **MreFreq** |
| --- | --- | --- | --- | --- | --- | --- | --- | --- |
| hsa-miR-571 | 163 | -23 | 163 | -23 | 21 | 258 | 113 | 1 |
| hsa-miR-34c-5p | 312 | -37.81 | 159 | -20 | 23 | 258 | 211 6 | 2 |
| hsa-miR-12119 | 311 | -48.27 | 163 | -26.48 | 25 | 258 | 231 95 | 2 |
| hsa-miR-4715-5p | 301 | -39.27 | 152 | -21.89 | 22 | 258 | 218 110 | 2 |
| hsa-miR-449a | 298 | -37.33 | 156 | -23.22 | 22 | 258 | 210 7 | 2 |
| hsa-miR-449b-5p | 298 | -36.88 | 156 | -20.9 | 22 | 258 | 210 7 | 2 |
| hsa-miR-6874-5p | 295 | -53.85 | 151 | -30.58 | 23 | 258 | 208 69 | 2 |
| hsa-miR-449c-5p | 294 | -40.68 | 148 | -24.88 | 25 | 258 | 5 208 | 2 |
| hsa-miR-6081 | 294 | -47.64 | 151 | -26.17 | 24 | 258 | 13 79 | 2 |
| hsa-miR-370-3p | 293 | -44.6 | 150 | -24.5 | 22 | 258 | 135 24 | 2 |

MiRLen, the length of miRNAs;CircLen, the length of circRNA

**Supplementary Table 3. The details of target genes of miR-34c-5p predicted by the TargetScan Database**

| **Gene Symbol** | **Position (3’UTR)** | **Site type** | **Context++ score** | **Context++ score percentile** | **Weighted context++ score** | **Conserved branch length** | **PCT** |
| --- | --- | --- | --- | --- | --- | --- | --- |
| IGSF1 | 274-281 | 8mer | -0.39 | 96 | -0.39 | 2.547 | 0.41 |
| CUEDC1 | 157-164 | 8mer | -0.49 | 98 | -0.49 | 4.933 | 0.88 |
| SGSM2 | 626-633 | 8mer | -0.35 | 94 | -0.35 | 4.902 | 0.88 |
| CDIP1 | 1248-1255 | 8mer | -0.48 | 98 | -0.48 | 2.884 | 0.59 |
| LGR4 | 1304-1311 | 8mer | -0.41 | 96 | -0.41 | 3.607 | 0.82 |

IGSF1, immunoglobulin superfamily member 1; CUEDC1, CUE domain containing 1; SGSM2, small G protein signaling modulator 2; CDIP1, cell death-inducing p53 target 1; LGR4, leucine-rich repeat containing G protein-coupled receptor 4.
