# Supplementary material for: Hsa_circ_0003945 promotes progression of hepatocellular carcinoma by mediating miR‐34c‐5p/LGR4/β‐catenin axis activity
Source: J Cell Mol Med. 2022 Feb 16;26(8):2218–29. doi: 10.1111/jcmm.17243 (PMC8995453; doi:10.1111/jcmm.17243)
Supplement: Supplementary file 2 — Supplementary Material [file JCMM-26-2218-s001.doc]

**Supplementary materials and methods**

**Total RNA isolation, reverse transcription, and quantitative real-time PCR (qPCR)**

Total RNA was isolated and extracted from HCC tissue and cells using EastepTM Super Total RNA Extraction Kit (Cat. LS1040, Promega, Madison, WI, USA) according to the manufacturer’s instructions. An equal amount of RNA was reverse transcribed into first-strand cDNA using a GoScript Reverse Transcription Mix kit (Cat. A2800, Promega). Primer sequences are shown in the Table 1 below. The level of circRNAs and related mRNAs in HCC tissue and cells was measured by qPCR using Go Taq® qPCR and RT-qPCR Systems (Cat. A6001, Promega). 18S rRNA was employed as endogenous control to measure the level of circRNAs and mRNAs. U6 was used as miRNA endogenous control. The amplification procedure was set up as follows: 95°C for 10 min followed by 40 cycles of 95°C for 15 s and 64°C for 30s. Melting curve analysis was used to confirm amplification specificity. The 2 ^(-ΔΔCt)^ method was used to evaluate circRNA levels.

**Table 1. The primer details used for RT-PCR**

| **Gene Symbol** | **Forward primer(5'-3')** | **Reverse primer(5'-3')** |
| --- | --- | --- |
| Circ_0001955 | GCCTATGATTGGGTTGGGAGA | GTTCGGTCACAGAGGTCAAAC |
| Circ_0003945 | GCCAAGCCCTTGTCTTCACA | GCAAGGCTACCAGATCAATGC |
| Circ_0005397 | TGCCGTTAACAACAAGCATTC | TGGAACTCTCTCTGGGGTGA |
| Circ_0027478 | CCATTGCCTGGAGTTGGCT | CCACAGCGTTTACAGAGTCG |
| Circ_0038718 | GTGGCAAGCTCTGGGAACAT | AGAGCAAAGCCACCCCATTG |
| CSPP1 | TGGCACTTCAGAAACGCTGA | CTGCCAAGTGAAAGTGCCTG |
| CSNK1G1 | CATCTGGCTCCTCATCGTCC | GAGCTCTCCGAAGTTCCCAC |
| UBAP2 | GCTGGTTCAAGGCTGTTTGG | AACAGGTGGCCATACTTGGG |
| RHOT1 | GTGCTCACTTGGCTCACTCT | AGACTGTGCGATGGCTTCAA |
| NUP107 | GGCAGGAACAGCTGGACATT | CGTACAACCTGGCAAAACCG |
| CircANRIL | GCTGGGATTACAGGTGTGAGACACC | GAATCAGAATGAGGCTTATTCTTCTCATC |
| IGSF1 | GCGTAGAGACACATCCTAACATC | TCCGACACTTCCACCTTATCC |
| CUEDC1 | CGACTCGGAGGACAGCATC | GCGTCTCTGGCTTGTAGGG |
| LGR4 | GGACCGCCGAGGAAGAGAC | GAAGCAGAGCAGCCCTAGC |
| SGSM2 | AGCAGGTGTTGGCAGAGTG | GGTGGAGTCTCGGTGGATG |
| CDIP1 | CCACAGTCCTGGTCCCTTC | GCACGAAATTCATCAAGCCAATC |
| c-Myc | GGCTCCTGGCAAAAGGTCA | CTGCGTAGTTGTGCTGATGT |
| CyclinD1 | CCCTCGGTGTCCTACTTCA | CTCCTCGCACTTCTGTTCCT |
| 18S rRNA | CAGCCACCCGAGATTGAGCA | TAGTAGCGACGGGCGGTGTG |
| U6 | CTCGCTTCGGCAGCACA | AACGCTTCACGAATTTGCGT |

CSNK1G1, casein kinase 1 gamma 1; UBAP2, ubiquitin associated protein 2; RHOT1, ras homolog family member T1; NUP107, nucleoporin 107; IGSF1, immunoglobulin superfamily member 1; CUEDC1, CUE domain containing 1; LGR4, leucine-rich repeat containing G protein-coupled receptor 4; SGSM2, small G protein signaling modulator 2; CDIP1, cell death-inducing p53 target 1; c-Myc, MYC proto-oncogene.

**RNase R treatment**

Total RNA (5 μg) extracted from HCC cells was incubated for 30 min at 37°C with or without 5 U/μg RNase R (Cat. RNR07250, Epicentre Technologies, Madison, WI, USA), followed by inactivation of RNase R at 70℃ for 10 min and reverse transcription of RNA using a random primer^1^.

**Actinomycin D treatment**

HCC cells were exposed to 2 μg/mL actinomycin D (Cat. 15021S, Cell Signaling Technology, Danvers, MA, USA) for 0, 4, 8, 12, or 24 h. Cells were then harvested, and total RNA was extracted. qPCR was performed to measure the stability of circRNAs and the associated mRNAs. Stability was normalized to the value measured in the group at 0 h^2,3^.

**Proliferation, wound healing, and Transwell migration assays**

The viability of HCC cells was measured using the CCK-8 Kit (Cat. C0039, Beyotime Biotechnology, China) following the manufacturer's instructions. Optical density was measured at 450 nm using a multifunctional microplate reader (Flex Station 3, Molecular Devices, San Jose, CA, USA). For the wound-healing assay, 5×10^5^ transfected cells in complete medium supplemented with 10% FBS were seeded in 24-well plates. When the cells reached confluence, a 200-μL sterile tip was used to scratch the monolayer at 0 h and the cells were photographed. Cells were then cultured in medium containing 1% FBS. Cells were observed and photographed 48 h later. For the Transwell assay, serum-free medium containing 3×10^4^ transfected cells was placed in the upper 24-well Transwell chambers and culture medium containing 10% FBS was added to the lower chambers. After incubation for 48 h, cells in the Transwell chambers were fixed using 4% paraformaldehyde followed by staining with 0.1% crystal violet. The migratory cells at the lower surfaces of chambers were photographed and counted.

**Subcellular fractionation**

Nuclear and cytoplasmic RNA were extracted using a Cytoplasmic and Nuclear RNA Purification Kit (Cat. 21000, Norgen, Thorold, Ontario, Canada). Briefly, HCC cells were lysed in lysis buffer on ice for 15 min, followed by centrifugation for 5 min at 12,000×g. The supernatant was collected to isolate cytoplasmic RNA, and the pellet was used to isolate nuclear RNA according to the manufacturer's instructions. qPCR was carried out to measure the relative expression level of circRNAs.

**Fluorescence in situ hybridization (FISH)**

RNA FISH probes for Circ_0003945, 18S rRNA, and U6 were designed and synthesized by RiboBio (Guangzhou, China). MHCC97H cells were seeded onto climbing sheets on the bottom of the 24-well plates. Cy3 probes specific to Circ_0003945, 18S rRNA, or U6 were applied to cells, and nuclei were counterstained with DAPI. The fluorescence in situ hybridization kit (Cat. C10910, RiboBio, Guangzhou, China) was used according to the manufacturer’s instructions. The probe signals were photographed using a Zeiss LSM800 Confocal Microscope system (Carl Zeiss AG, Oberkochen, Germany).

**Plasmid construction and transfection**

To construct Circ_0003945 stable-knockdown HCC cell lines, small hairpin RNA (shRNA) specifically targeting the junction region of the Circ_0003945 sequence (CAGAGCCTGCTTTCTGAAA) was designed and synthesized. Vector pLshRNA-NC was used to construct a shRNA plasmid. Meanwhile, the sequence of cDNA encoding human Circ_0003945 was synthesized and cloned into vector pLC5-ciR to generate overexpression plasmids. The constructed plasmids were then transfected into HEK293T cells to package the lentivirus and infect HCC cells. Finally, cells were treated with 2 μg/mL puromycin to construct stable transfectants. The negative control oligos for miRNAs, miRNA mimics, and miRNA inhibitors were purchased from GenePharma (Shanghai, China). Lipofectamine 3000 (Invitrogen, Carlsbad, CA, USA) was used to transfect cells according to the manufacturer’s instructions.

**RNA immunoprecipitation (RIP) assay**

HCC cells were treated with lysis reagent containing RNase inhibitor and proteinase inhibitors, and then anti-argonaute-2 (AGO2) antibody (Cat. 2897, Cell Signaling Technology) was added to the lysate and incubated overnight. Next, agarose beads were used to co-precipitate the RISC-Ago2 complex, followed by washing with lysis buffer four times. Finally, the co-precipitated RNA was purified using TRIzol reagent (Thermo Fisher Scientific, Waltham, MA, USA) and analyzed by qPCR, with normal mouse IgG as the negative control.

**Bioinformatics analysis**

According to previous studies, the CircBank^4^, CircInteractome^5^ and miRanda (https://www.winsite.com/Multimedia/Image-Editors/Miranda-Password-Recovery/) online databases were used to predict the miRNA targets of Circ_0003945. The TargetScan^6^ and miRDB online databases were utilized to predict the candidate targets of miR-34c-5p.

**Luciferase reporter assay**

To evaluate potential binding between miR-34c-5p and Circ_0003945, wild-type sequences containing the binding sites of miR-34c-5p in Circ_0003945 or mutant sequences were inserted into the vector psiCHECK2. Further, vector pGL3 was used to construct wild-type and mutant plasmids to verify the correlation between miR-34c-5p and LGR4. Subsequently, HEK-293T cells were seeded into 24-well plates at a density of 1 × 10^4^ cells per well. The luciferase reporter (1 μg) and hsa-miR-34c-5p mimics (50 nM) or hsa-miR-571 mimics (50 nM), or negative control miR-NC (50 nM) were co-transfected into HEK-293T cells. After 48 h, luciferase activity was detected using the Dual Luciferase Reporter Assay System (Cat. E1910, Promega, Madison, WI, USA).

**Western blotting**

Briefly, protein was extracted from cells in RIPA lysis buffer (Cat. P0013B, Beyotime, Shanghai, China) containing a protease inhibitor cocktail for general use (Cat. P1009, Beyotime, Shanghai, China) and protease and phosphatase inhibitor cocktail (Cat.P1050, Beyotime, Shanghai, China), according to the manufacturer's protocols. Equal amounts of total protein (20 μg) were loaded onto a 10% SDS–PAGE gel, separated, and transferred to polyvinylidene fluoride (PVDF) membranes (Cat. FFP36, Beyotime, China). Bovine serum albumin (5%) was suspended in 1× Tris-buffered saline with Tween (TBST) and used to block nonspecific binding followed by incubation with anti-UBAP2 (1:1000, Cat. A304-626A, Thermo Fisher Scientific), anti-LGR4 (1:1000, Cat. ab137480, Abcam, Cambridge, UK), anti-tubulin (1:1000, Cat. 2148, Cell Signaling Technology), anti-β-catenin (1:1000, Cat. 8480S, Cell Signaling Technology), anti-phospho–β-catenin (1:1000, Cat. 9561, Cell Signaling Technology), anti-histone H3 (1:1000, Cat. 4499, Cell Signaling Technology), anti-GAPDH (1:1000, Cat. AF5009, Beyotime, China), anti-c-Myc (1:1000, Cat. 5605S, Cell Signaling Technology) and anti-CyclinD1 (1:1000, Cat. 2978S, Cell Signaling Technology) antibodies overnight at 4℃. After three washes of the PVDF membranes with 1× TBST, membranes were incubated with secondary antibody for 2 h at room temperature (1:1000, Cat. A0216 or A0208, Beyotime, China). The protein bands were visualized through a chemiluminescent reaction (Cat. P0018FM, ECL, Beyotime, China). Image Lab software (Bio-Rad, Hercules, CA, USA) was used to perform semi-quantitative analysis.

**Immunohistochemical (IHC) staining**

The mouse xenograft tumors were embedded in paraffin, 5-μm thick sections were prepared, placed on glass slides, and stained with hematoxylin and eosin (H&E) and processed for IHC analysis. The slides underwent deparaffinization, rehydration, antigen retrieval, and incubation at 4℃ overnight with primary antibodies against Ki-67 according to the manufacturer's instructions. The next day, sections were incubated with horseradish peroxidase-conjugated goat anti-rabbit IgG at room temperature for 1 h followed by staining with diaminoaniline and counterstaining with hematoxylin. Finally, the slides were sealed with coverslips used for the fluorescence microscope. Images were captured using the Olympus IX71 system with cell Sens Standard software (Olympus, Tokyo, Japan).

**References:**

1 Cheng, Z. F. & Deutscher, M. P. Purification and characterization of the Escherichia coli exoribonuclease RNase R. Comparison with RNase II. J BIOL CHEM 277 21624 (2002).

2 Zheng, Q., Bao, C., Guo, W., Li, S., Chen, J. & Chen, B. et al.. Circular RNA profiling reveals an abundant circHIPK3 that regulates cell growth by sponging multiple miRNAs. NAT COMMUN 7 11215 (2016).

3 Yu, J., Xu, Q. G., Wang, Z. G., Yang, Y., Zhang, L. & Ma, J. Z. et al.. Circular RNA cSMARCA5 inhibits growth and metastasis in hepatocellular carcinoma. J HEPATOL 68 1214 (2018).

4 Glazar, P., Papavasileiou, P. & Rajewsky, N. circBase: a database for circular RNAs. RNA 20 1666 (2014).

5 Dudekula, D. B., Panda, A. C., Grammatikakis, I., De S, Abdelmohsen, K. & Gorospe, M. CircInteractome: A web tool for exploring circular RNAs and their interacting proteins and microRNAs. RNA BIOL 13 34 (2016).

6 Agarwal, V., Bell, G. W., Nam, J. W. & Bartel, D. P. Predicting effective microRNA target sites in mammalian mRNAs. ELIFE 4 (2015).

7 Liu, W. & Wang, X. Prediction of functional microRNA targets by integrative modeling of microRNA binding and target expression data. GENOME BIOL 20 18 (2019).
